# Innovative zero-valent cobalt decoration on MIL-88 A(Fe)@β-CD for high-efficiency and reusable cr(VI) removal

**DOI:** 10.1038/s41598-025-88259-y

**Published:** 2025-02-05

**Authors:** Eman M. Abd El-Monaem, Nouf Al Harby, Mervette El Batouti, Abdelazeem S. Eltaweil

**Affiliations:** 1 Advanced Technology Innovation, Borg El-Arab, Alexandria Egypt; 2 Department of Chemistry, College of Science, Qassim University, 51452 Buraidah, Saudi Arabia; 3 Department of Chemistry, Faculty of Science, Alexandria University, Alexandria, Egypt; 4 Department of Engineering, College of Engineering and Technology, University of Technology and Applied Sciences, Ibra, Sultanate of Oman

**Keywords:** Mechanistic study, MIL-88A, ZVCo decoration, β-CD, Kinetic assessments, Reusability, Environmental sciences, Chemistry

## Abstract

Herein, the magnetic ZVCo-MIL-88 A(Fe)@β-CD composite was fabricated via post-synthetic decoration of MIL-88 A(Fe)@β-CD with ZVCo to produce a magnetic efficient adsorbent for Cr(VI) removal. The experimental findings denoted that the ZVCo decoration boosted the adsorption capability of MIL-88 A(Fe)@β-CD, where the adsorption % of Cr(VI) improved from 73.07 to 94.02% after its decoration with 10 wt% of ZVCo. Furthermore, the ZVCo decoration ameliorated the recycling feature of MIL-88 A(Fe)@β-CD since the removal % of Cr(VI) by MIL-88 A(Fe)@β-CD and ZVCo-MIL-88 A(Fe)@β-CD reached 27.24 and 84.98%, respectively. The optimization experiments of the Cr(VI) ions clarified that the higher adsorption % fulfilled 94.02% at pH = 3, using ZVCo-MIL-88 A(Fe)@β-CD dosage = 0.5 g/L, Cr(VI) concentration = 50 mg/L, and at room temperature. Notably, the concentration of the adsorbed Cr(VI) brings off the equilibrium stage within an hour, implying the fast adsorption property of ZVCo-MIL-88 A(Fe)@β-CD. The kinetic and isotherms assessments denoted the contribution of the physical and chemical adsorption pathways in adsorbing the Cr(VI) species onto ZVCo-MIL-88 A(Fe)@β-CD. In addition, the XPS spectra and zeta potential results supposed that the process inside the Cr(VI)/ZVCo-MIL-88 A(Fe)@β-CD system proceeded through reduction reaction, coordination bonds, electrostatic interactions, and pore-filling mechanisms.

**Supplementary Information:**

The online version contains supplementary material available at 10.1038/s41598-025-88259-y.

## Introduction

The development in the bountiful of heavy and light industries brings many environmental losses. Even though industrialization development prospers the economy around the world, it results in forming different kinds of pollution, such as noise, air, water, and soil. Water pollution is a serious affair that can destroy the earth entirely, as it affects humans, the environment, and other living organisms. Various pollutants are severe in wastewater, like radioactive substances, trace elements, organic/inorganic pollutants, sewage, and sediments^1–5^.

Chromium is one of the diffused trace elements in water bodies that is deemed the core of gigantic industries, such as mining, electroplating, leather, pigment, and metal cleaning; nevertheless, chromium is listed at the top of the most dangerous sixteen heavy metals^6–8^. There are two most common valences of chromium: trivalent (Cr(III)) and hexavalent (Cr(VI)), where Cr(III) is a benign form, and Cr(VI) is a detrimental form^9^. Therefore, reducing Cr(VI) to Cr(III) can be a functional removal pathway of the notorious Cr(VI)^10^. Remedying wastewater from the Cr(VI) species proceeds with various techniques, including ozonation, catalysis, membranes, adsorption, and precipitation^11–13^. Interestingly, the adsorption technique provides individual merits like costless, energy saving, high efficiency, eco-benign, and facile processing^14,15^. Consequently, frequent studies per year involve developing adsorbents to have high adsorption aptitude and excellent recycling properties.

Metal-organic frameworks (MOFs) are functional substances that have exhibited promising results in remedying wastewater from sundry contaminants^16,17^. The MOF materials are set up by forming a coordination bond between unsaturated metal species and organic linker^18^. Impressively, pioneering investigations regarding MOFs confirmed their excellent performances in diffusing, transporting, and trapping the targeted contaminants owes to their superb features, comprising good supporter, large surface area, stability in water, porosity, flexibility, and pre-eminent adsorption aptitude^19,20^.

MIL-88 A(Fe) is a 3D-topology MOF that is synthesized by hydro(solvo)-thermal reaction between the ferric species and fumaric acid^21^. The used solvent in fabricating the MIL-88 A(Fe) identifies its morphology since its shape is hexagonal when the used solvent N, N-dimethylformamide while the utilizing of water as a solvent yields MIL-88 A(Fe) with a rod shape^22^. N, N-dimethylformamide is a highly toxic solvent, which is classified among the carcinogenic solvents. Noteworthy, N,N-dimethylformamide can dwell in the network of MOFs, giving rise to forming a secondary contaminant. So, N,N-dimethylformamide-free MOFs need further investigation and development, using benign solvents like water instead of N, N-dimethylformamide^23^. Nevertheless, MIL-88 A(Fe), like MOFs, suffers from a main shortcoming: the high dispersity that obstacles its separation from the adsorption medium and thus declines its recyclability. Hence, shaping the MOFs into membranes and beads or fabricating magnetic MOF-based composites could be the viable solution to conquer this bottleneck of MOFs^24^.

Magnetic nanoparticles offer outstanding advantages, such as tiny particle size, high adsorption efficacy, chemo-selectivity, and excellent recyclability, making them a promising choice as propitious adsorbents^25,26^. Zero-valent cobalt (ZVCo) are magnetic nanoparticles, having individual features like the high super-paramagnetic that endows them with high recycling and easy-separation properties^27^. Furthermore, ZVCo, likewise the ZV-metals, can in situ produce Co^2+^ to participate in the adsorption mechanism via the reduction reaction^28^. Although the magnetic property of ZVCo gives it the perfect and easy separation merits, it causes particle aggregation that decreases the available surface area. So, blinding the highly magnetic ZVCo with non-magnetic substances could be a feasible strategy to outdo the aggregation problem of the magnetic particles of ZVCo.

The cyclic oligosaccharide cyclodextrin (CD) is a cone-shaped topology constructed from a network of the macro glucopyranose ring^29^. There are three forms of CD, which are γ-CD, β-CD, and α-CD that contain eight, seven, and six glucopyranose rings, and their cavities’ diameters are 0.75–0.83, 0.6–0.65, and 0.47–0.53 nm, respectively^30–32^. Owing to the facile preparation and favorable cavity diameter of β-CD, it is widely applied compared to the other CD forms^33^. The structure of CD looks like a host-guest relationship, where the host is a hydrophobic construct of the CH_2_ group, and the guest is hydrophilic, comprising branches of the OH group^34^. The flexible architecture of CD endows it with the easy-functionalization feature, improving its chemical and physical properties^35,36^. Furthermore, the large surface area, biocompatibility, sustainable production, and porous structure of CD render it a good adsorbent since it can capture organic/inorganic pollutants from wastewater^37^. Nonetheless, CD’s good solubility limits its applicability in water remediation. The fabrication of CD-based composites could solve this issue since the large numbers of OH in the CD matrix can effectively capture contaminants from wastewater and interact with other functional groups to form composites^38^.

In this regard, Kekes et al. fabricated chitosan/β-CD beads for removing Cr(VI), deducing that the maximum adsorption capacity (Q_max_) of Cr(VI) was 555.56 mg/g at pH = 4, temperature = 15 °C, and beads dose of 20 g/L^39^. Moreover, Wang and his co-authors modified β-CD with magnetic graphene oxide (m-GO) to efficiently adsorb Cr(VI). The experimental results denoted that the calculated Q_max_ of Cr(VI) under Langmuir onto β-CD/m-GO was 49.95 mg/g at pH = 2. In addition, the removal % of Cr(VI) by β-CD/m-GO was 73% after five adsorption runs^40^. In another investigation, Wang et al. fabricated β-CD/polyaniline composite for removing Cr(VI), elucidating that the higher removal % of Cr(VI) attained 98% at pH = 6 via coupling between adsorption and reduction mechanisms^41^. Furthermore, Hammad et al. highlighted the modification of cetyl pyridinium bromide-functionalized MIL-88 A by aminated graphene oxide (CPBr-MIL-88 A@AmGO). The Q_max_ of Cr(VI) onto CPBr-MIL-88 A@AmGO reached 306.75 mg/g at pH = 2. Studying the influence of coexisting cations, such as Na^+^, K^+^, and Ca^2+^, and anions like Cl^−^ and NO_3_^−^ on the Cr(VI) adsorption revealed an inconsiderable effect on the efficacy of the adsorption process. At the same time, anions like SO_4_^2−^ in the adsorption medium could hinder the adsorption of Cr(VI) onto CPBr-MIL-88 A@AmGO^42^. Kakavandi et al. synthesized by supporting zerovalent iron/silver on activated carbon (ZVFe/Ag@AC). The Q_max_ of Cr(VI) onto ZVFe/Ag@AC was 100 mg/g at pH = 3. In addition, the adsorption of Cr(VI) onto ZVFe/Ag@AC fulfilled equilibrium within an hour^43^. In this context, Qiu et al. deduced that the adsorption mechanism of Cr(VI) onto the ZVFe-supported biochar composite proceeded via direct/ortho reduction and electrostatic attraction. Furthermore, the higher adsorption % of Cr(VI) was 64.13% at pH = 4, reaction temperature = 25 °C, and dose = 1 g/L^44^.

The recent advancements in fabricating efficient adsorbents to remove Cr(VI) from wastewater have focused on exploiting the advantage of bountiful advanced materials like MOFs, zerovalent metals, and CDs, as abovementioned in the literature survey. In contrast, there are limitations in the research papers regarding the utilization of some types of MOFs like MIL-88 A and zerovalent metals such as ZVCo for the adsorptive removal of Cr(VI).

Our study highlighted developing an efficacious and recyclable adsorbent to remove the lethal Cr(VI) ions from wastewater by decorating MIL-88 A(Fe)@β-CD surface with ZVCo to produce the magnetic ZVCo-MIL-88 A(Fe)@β-CD composite. The batch experiments proceeded to record the favorable conditions to adsorb Cr(VI), including the optimal pH, temperature, equilibrium time, and adsorbent dose. Then, the resultant data from the experimental work were analyzed by kinetics and isotherms models to deduce the governing interaction’s type. Furthermore, the physical forces and the chemical interactions between Cr(VI) and ZVCo-MIL-88 A(Fe)@β-CD composite were supposed in light of the XPS spectra. Competitive anionic species like nitrate, chloride, and sulfate were introduced to the Cr(VI)/ZVCo-MIL-88 A(Fe)@β-CD system to imitate actual wastewater. More importantly, to demonstrate the durability and applicability of the ZVCo-MIL-88 A(Fe)@β-CD composite, recycling, and leaching tests were conducted.

## Experimental section

The materials that were utilized to fabricate ZVCo-MIL-88 A(Fe)@β-CD composite and the characterization apparatus are summarized in Text S1 and S2.

### Fabricating the ZVCo nanoparticles

The ZVCo nanoparticles were prepared via the reduction approach as follows: dissolving 180 mg of CoCl_2_.6H_2_O in mixed solvents of double distilled water (3 mL) and Et-OH (7 mL). In another container, 420 mg of NaBH_4_ was dissolved in 33 mL of double distilled water. The obtained aqueous solution of NaBH_4_ was rapidly dropped on the Co^2+^ solution to decline the reaction time, avoiding the oxidation of the produced ZVCo. For complete growth, the ZVCo nanoparticles were stirred potently for 5 min, then they were collected by a magnet, washed with Et-OH, and dried for 12 h at 50 °C^45^.

### Fabricating MIL-88 A(Fe)

The rod-shaped MIL-88 A(Fe) was synthesized by the hydrothermal method as follows: adding 2.6 g of FeCl_3_·6H_2_O and 1.2 g of Fumaric acid (F. Acid) in 65 mL of double distilled water with a continuous stirring for 2 h until complete homogeneity of the metal-linker solution. Then, the bright-orange solution of Fe^2+^/F. Acid was poured into the autoclave and kept in the oven for a day at 80 °C. Next, after cooling the autoclave at room temperature, the MIL-88 A (Fe) was collected via centrifugation, washed with double distilled water and Et-OH, and heated in an oven at 70 °C for 12 h until utter drying^46^.

### Fabricating ZVCo-MIL-88 A(Fe)@β-CD composite

The ZVCo-MIL-88 A(Fe)@β-CD composite was prepared via the post-synthetic approach as follows: adding 0.05 g of MIL-88 A(Fe) and 0.025 g of β-CD in 25 mL of double distilled water with continuous stirring for an hour, following by sonication for another one hour to form a homogeneous black solid of the ZVCo-MIL-88 A(Fe) composite. Next, ZVCo (5–20 wt%) was distributed in the ZVCo-MIL-88 A(Fe) suspension, completing the sonication for an hour. Finally, the ZVCo-MIL-88 A(Fe)@β-CD composite was dried at 70 °C for 12 h.

### Batch experiments

Sequential adsorption experiments proceeded to record the best conditions to adsorb the Cr(VI) onto the ZVCo-MIL-88 A(Fe)@β-CD composite. (i) Identifying the appropriate amount of the ZVCo in the ZVCo-MIL-88 A(Fe)@β-CD composite by performing a series of the Cr(VI) adsorption experiments using the composites with different ZVCo proportions. (ii) investigating the suitable pH medium by changing the pH of the Cr(VI)/ZVCo-MIL-88 A(Fe)@β-CD system within the range of 3–11. (iii) determining the ideal dosage of ZVCo-MIL-88 A(Fe)@β-CD for adsorbing the Cr(VI) ions, altering the composite dosage from 0.25 to 1.00 g/L. (iv) recording the equilibrium time and the isotherms of the Cr(VI) adsorption onto ZVCo-MIL-88 A(Fe)@β-CD at varies concentrations of the Cr(VI) species. (v) realizing the thermodynamics of the Cr(VI)/ZVCo-MIL-88 A(Fe)@β-CD system by elevating the process temperature up to 55 °C. (vi) assessing the impact of the competitive co-interfering anions by introducing sulfate, chloride, and nitrate ions between the concentrations 10–30 mM to the Cr(VI)/ZVCo-MIL-88 A(Fe)@β-CD system. (vii) confirming the durability of the ZVCo-MIL-88 A(Fe)@β-CD composite by the recycling test for five runs using methanol/NaOH as the eluent. The initial (C_i_) and final (C_f_) concentrations of the Cr(VI) species were identified via spectrophotometer, and the Eqs. (1, 2) were applied to investigate the aptitude of the Cr(VI) adsorption^47,48^.

1 $$\:Removal\:\%,\:R\:\left(\%\right)=\frac{{C}_{i\:}-{C}_{f}}{{C}_{i}}\:\times\:100$$

2 $$\:Adsorption\:capacity,\:Q\:(mg/g)=\frac{{(C}_{i}-{C}_{f})\times\:V}{m}$$

## Results and discussion

### Characterization of ZVCo-MIL-88 A(Fe)@β-CD

#### FTIR spectra

**Fig. 1:**
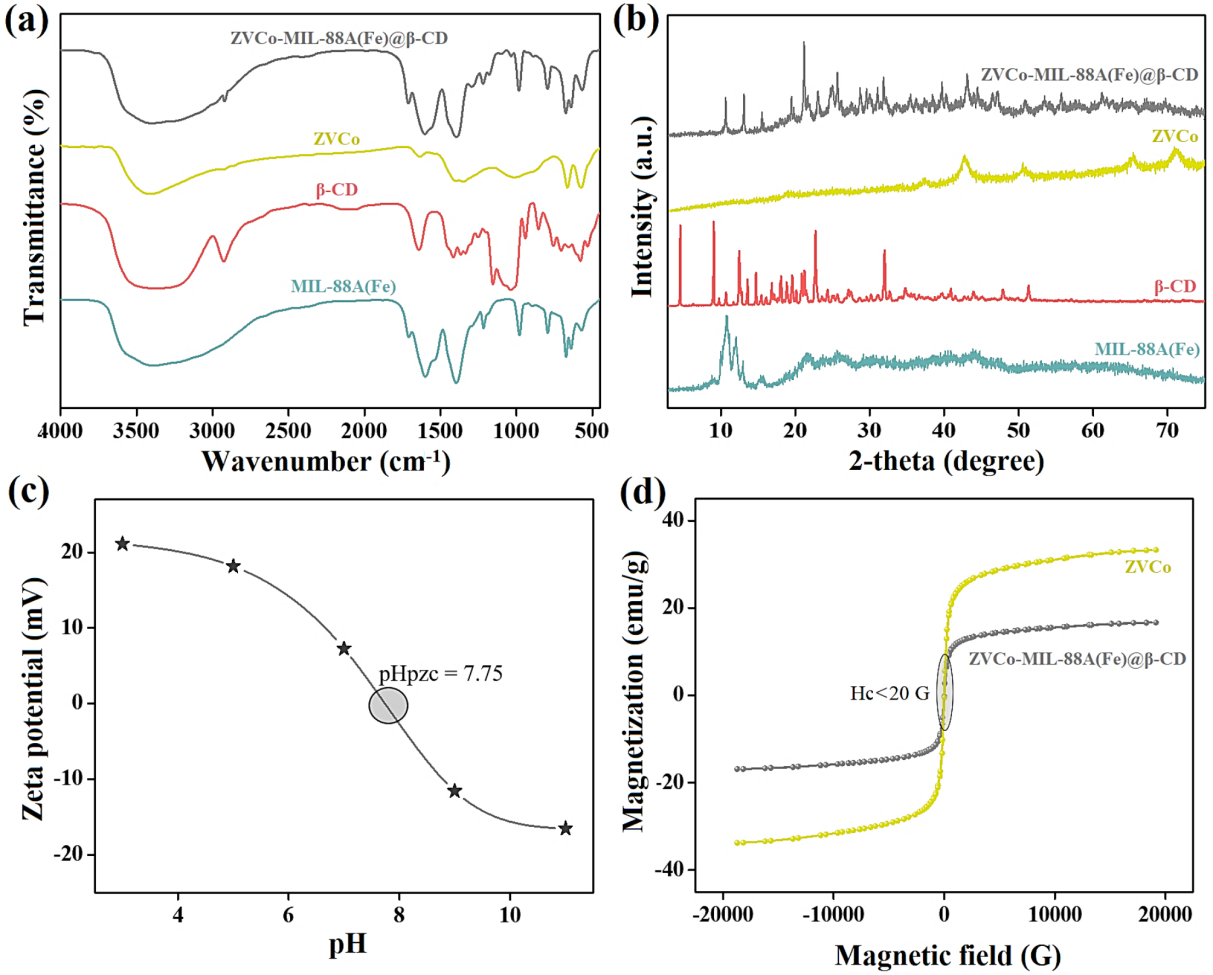
(**a**) FTIR spectra and (**b**) XRD pattern of ZVCo, MIL-88 A(Fe), β-CD, and ZVCo-MIL-88 A(Fe)@β-CD, (**c**) Zeta potential curve of ZVCo-MIL-88 A(Fe)@β-CD, and (**d**) The magnetization hysteresis loops of the neat ZVCo and ZVCo-MIL-88 A(Fe)@β-CD.

The compositions of ZVCo, MIL-88 A(Fe), β-CD, and ZVCo-MIL-88 A(Fe)@β-CD were confirmed by the FTIR analysis, as represented in Fig. 1a. For MIL-88 A(Fe), the FTIR pattern elucidated related peaks to the asymmetric carboxylate group of F. Acid at 1604 cm^−1^, while the symmetric carboxylate appeared at 1396 cm^−1^. Furthermore, the characteristic peak of Fe-O manifested at 573 cm^− 1^, evincing the formation of the coordination bond between the ferric ions and F. Acid^49^. The peaks at 1705 and 1716 cm^− 1^ correspond to C = O and C-C, and the distinguishing peaks to O-H and C-H appeared at 3374 cm^− 1^, respectively^50^. For β-CD, the FTIR pattern clarified the distinctive peaks to the linked α-1,4-glycosidic bonds between glucopyranose units^51^. The C-O vibrating peak presented at 1153 cm^− 1^, and the C-O stretching peak appeared at 1251 cm^− 1^. The C-H bending peaks manifested at 766, 1365, 1414, and 1365 cm^− 1^, while the C-H stretching peak evidenced at 2925 cm^− 1^. The absorption peaks at 1642 and 3363 cm^− 1^ are related to the OH bending vibrational and stretching vibrational, respectively. For ZVCo, the FTIR pattern showed peaks at 574 and 664 cm^− 1^, which assigns to the Co-O, and the peaks between 1000 and 1600 cm^− 1^ belong to the B-O vibration^52^. The low intensity of the FTIR pattern of ZVCo may be due to its amorphous structure^45^. For ZVCo-MIL-88 A(Fe)@β-CD, the FTIR revealed the combination between the absorption peaks of ZVCo, MIL-88 A(Fe), and β-CD, indicating the successful fabrication of the composite.

#### PXRD patterns

The PXRD diffractograms of ZVCo, MIL-88 A(Fe), β-CD, and ZVCo-MIL-88 A(Fe)@β-CD are illustrated in Fig. 1b. For MIL-88 A, the PXRD diffractogram demonstrated the distinguishing peaks at 2θ of 10.77°, 11.98⁰, 12.94⁰, 15.37⁰, and 21.42⁰ accompanied by the planes of (100), (101), (110), (012), and (022)^53,54^. For β-CD, the PXRD diffractogram represented the characteristic peaks at 2θ of 6.29°, 8.8°, 9.76°, 10.69°, 11.69°, 12.47°, 14.70°, 17.15°, 21.19°, 22.69°, 24.34°, 27.14°, 35.9°, and 41.47 correspond to the planes of (001), (101), (011), (130), (041), (141), (090), (042), (251), (162), (222), (223), and (044)^55–57^. For ZVCo, the PXRD diffractogram revealed the amorphous character of ZVCo with diffraction peaks at 2θ of 44.42°, 51.29°, 62.35°, and 72.11° signify the planes of (002), (101), (102), and (110)^58^. For ZVCo-MIL-88 A(Fe)@β-CD, the PXRD diffractogram showed the blinding between ZVCo, MIL-88 A(Fe), and β-CD with a decline in the peak intensity owes to the amorphous character of ZVCo.

#### Zeta potential measurements

Figure 1c exhibits the zeta potential of the ZVCo-MIL-88 A(Fe)@β-CD composite at different pHs between 3 and 11. The zeta potential versus pH plot of ZVCo-MIL-88 A(Fe)@β-CD demonstrated that the zero-charge point of the composite surface was at pH = 7.75. Consequently, the ZVCo-MIL-88 A(Fe)@β-CD surface charged with high positive charges in acidic media, reaching its peak at pH = 3, in which the zeta potential was 21.11 mV. On the contrary, ZVCo-MIL-88 A(Fe)@β-CD carried negative charges at alkaline media. These observations implied that ZVCo-MIL-88 A(Fe)@β-CD can adsorb the anionic pollutants in acidic media, and the cationic contaminants can be adsorbent onto the composite surface in alkaline media.

#### VSM hysteresis loops

The magnetization hysteresis loops of the neat ZVCo and ZVCo-MIL-88 A(Fe)@β-CD are clarified in Fig. 1d. The nitrogen isotherms revealed super-paramagnetic hysteresis loops of ZVCo and ZVCo-MIL-88 A(Fe)@β-CD, in which their coercivity magnitudes were 18.38 and 13.69 G, sequentially. This finding gives the magnetic ZVCo-MIL-88 A(Fe)@β-CD composite an interesting merit since the super-paramagnetic does not aggregate at room temperature after removing the external magnetic field^59^. Moreover, the saturation magnetization of ZVCo and ZVCo-MIL-88 A(Fe)@β-CD were 33.25 and 17.19 emu/g, respectively. The decline in the saturation magnetization of ZVCo is most likely due to the non-magnetization characters of MIL-88 A(Fe) and β-CD. Even though the diminution in the magnetization property of ZVCo after mixing with MIL-88 A(Fe) and β-CD in the composite, the magnetization of the composite is still sufficient to separate the composite utilizing an external magnet instead of filtration and centrifugation, providing a perfect separation in a short time.

#### SEM images

**Fig. 2:**
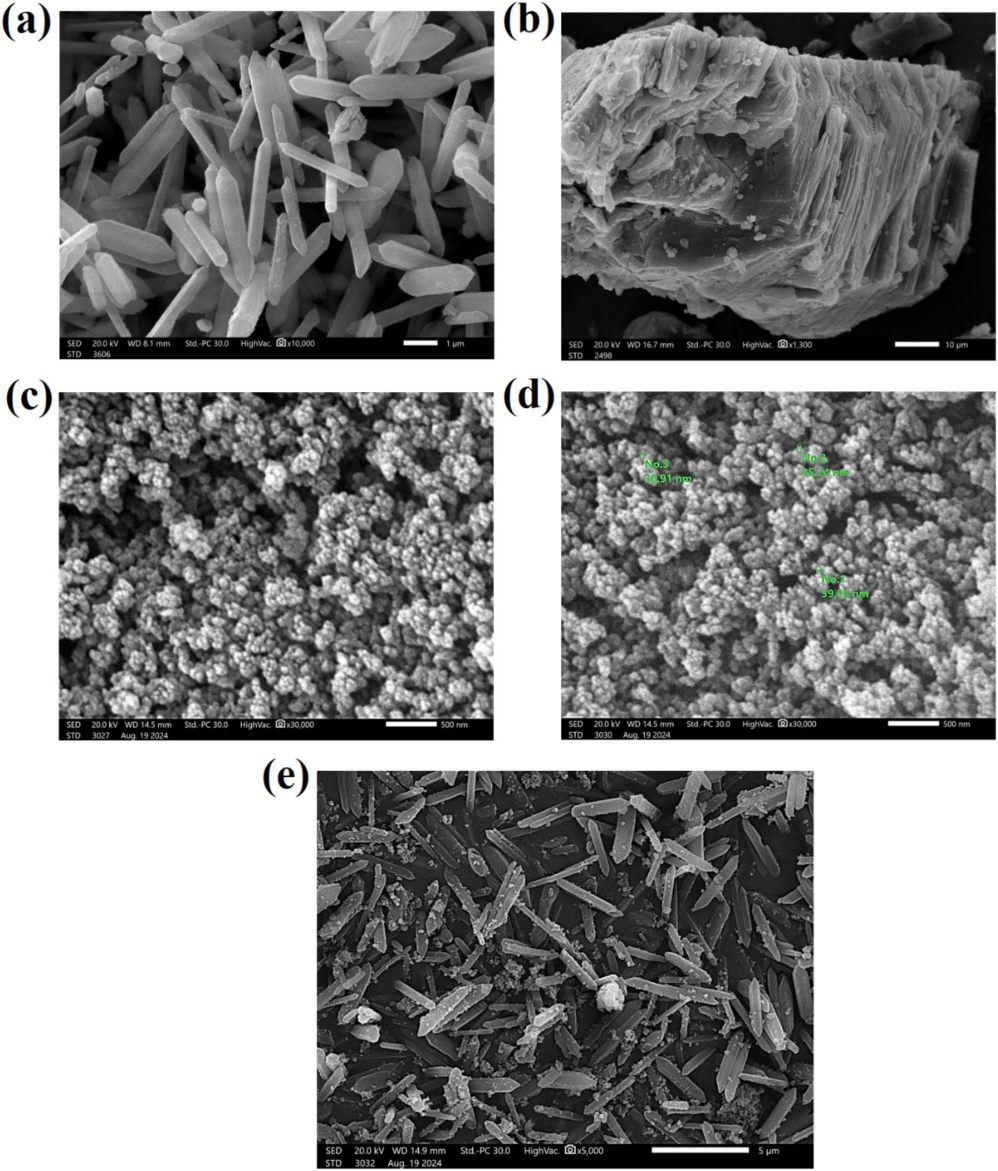
The SEM images of (**a**) MIL-88 A, (**b**) β-CD, (**c**,**d**) ZVCo, and (**e**) ZVCo-MIL-88 A(Fe)@β-CD.

The morphological shapes of ZVCo, MIL-88 A(Fe), β-CD, and ZVCo-MIL-88 A(Fe)@β-CD are elucidated in Fig. 2a–d. For MIL-88 A(Fe), the SEM image showed the typical rod shape formed by using the hydrothermal synthesization approach. The size of the MIL-88 A(Fe) rods was not uniform, where their length ranged between 2.5 and 4.5 µ and the width range was about 0.25–0.89 µ. For β-CD, the SEM image clarified its sheet-shaped morphology, and the surface of the sheets is rough. These characteristics of β-CD make it a suitable scaffold to hold highly distributed substances, such as MIL-88 A(Fe), and ease its separation. Furthermore, supporting the magnetic particles on the β-CD surface diminishes the aggregation of the particles and increases their surface area. For ZVCo, the SEM image depicted that the outer shape of the particles looks like a spherical shape with a tiny size in the nano range of 30.91 to 39.34 nm. For ZVCo-MIL-88 A(Fe)@β-CD, the SEM image represented the well-distribution of ZVCo and MIL-88 A(Fe) on the β-CD sheets, confirming working β-CD as a supporter.

#### BET measurements

**Fig. 3:**
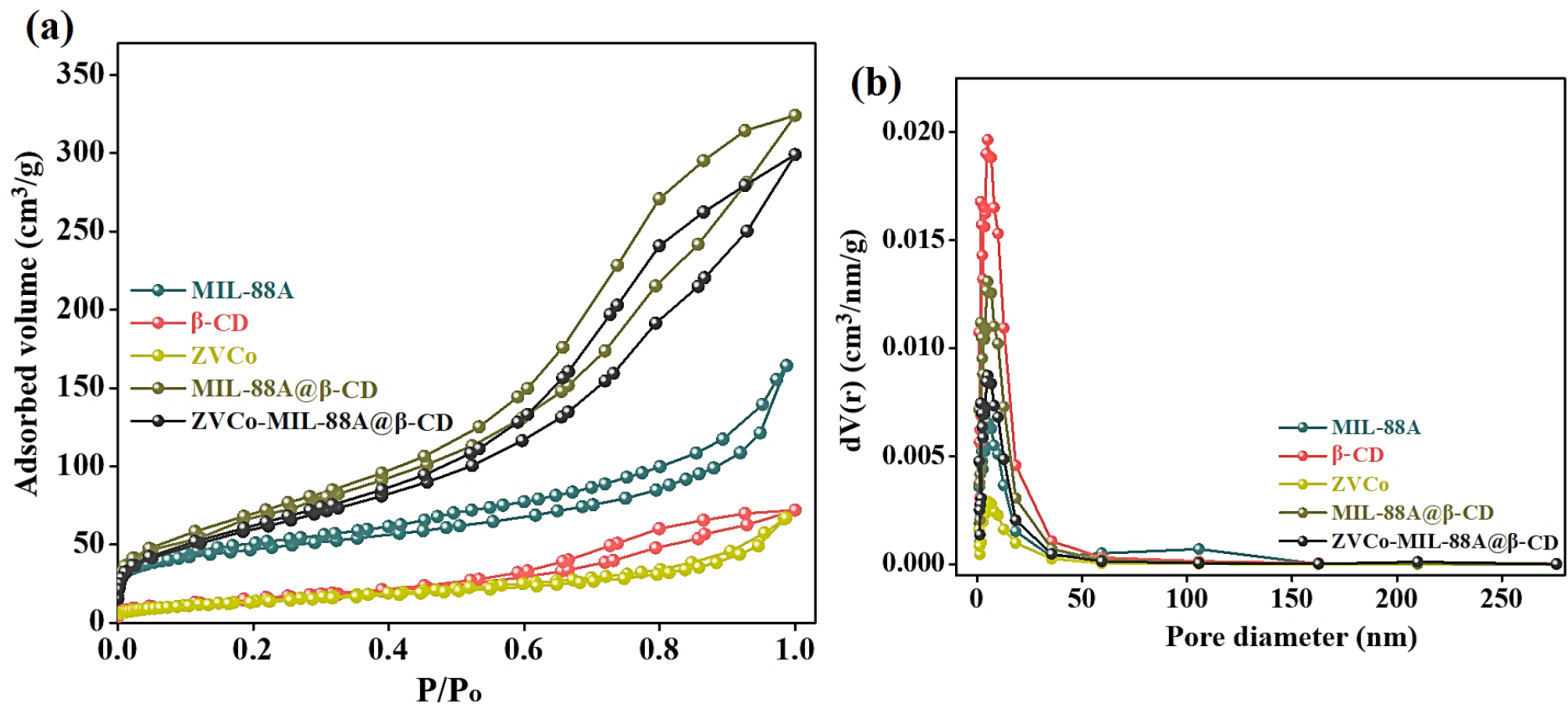
BET measurements of MIL-88 A(Fe), β-CD, ZVCo, MIL-88 A(Fe)@β-CD, and ZVCo-MIL-88 A(Fe)@β-CD; (**a**) N_2_ adsorption/desorption hysteresis loop and (**b**) BJH pore size distribution plot.

The nitrogen adsorption/desorption isotherms of MIL-88 A(Fe), β-CD, ZVCo, MIL-88 A(Fe)@β-CD, and ZVCo-MIL-88 A(Fe)@β-CD are illustrated in Fig. 3a. The isotherms of MIL-88 A(Fe), β-CD, ZVCo, MIL-88 A(Fe)@β-CD, and ZVCo-MIL-88 A(Fe)@β-CD depicted type (IV) with specific surface area values of 152.74, 54.15, 66.32, 244.13, and 216.34 m^2^/g, respectively. The BJH pore diameters of MIL-88 A(Fe), β-CD, ZVCo, MIL-88 A(Fe)@β-CD, and ZVCo-MIL-88 A(Fe)@β-CD were 2.15, 1.77, 7.99, 6.44, and 5.29 nm, as depicted in Fig. 3b. The BET measurements suggested the mesoporous structures of MIL-88 A(Fe), β-CD, ZVCo, MIL-88 A(Fe)@β-CD, and ZVCo-MIL-88 A(Fe)@β-CD. Additionally, the slight decline in the surface area of MIL-88 A(Fe)@β-CD after doping with ZVCo is attributed to the super-paramagnetic nature that causes particle aggregation and increases the particle size, which diminishes the surface area.

#### XPS spectra

**Fig. 4:**
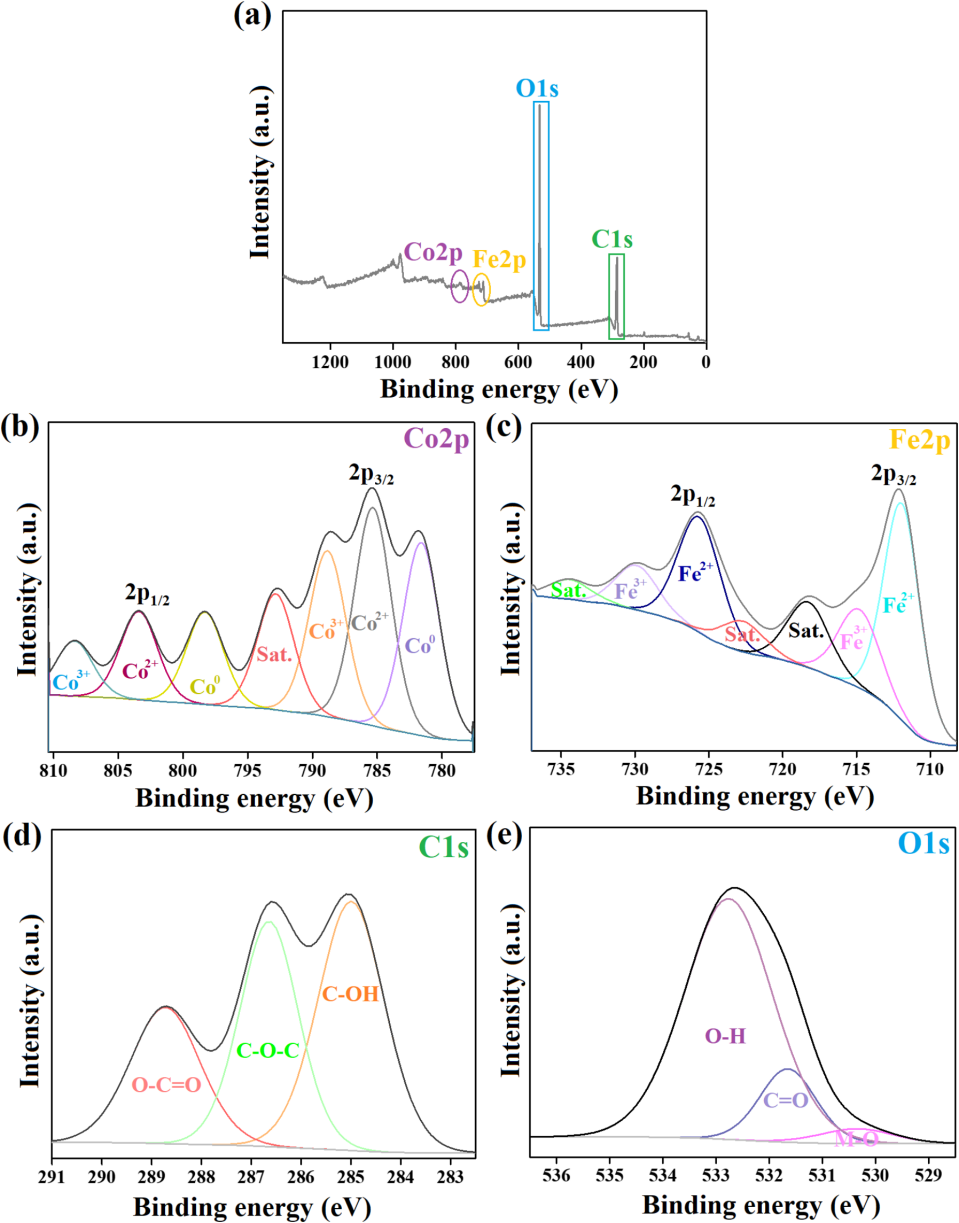
The XPS spectra of ZVCo-MIL-88 A(Fe)@β-CD; (**a**) Wide-spectrum, (**b**) Co2p, (**c**) Fe2p, (**d**) C1s, and (**e**) O1s.

The elemental composition of the ZVCo-MIL-88 A(Fe)@β-CD composite was investigated by the XPS analysis, as depicted in Fig. 4a–e. The wide-spectrum elucidated the composition of ZVCo-MIL-88 A(Fe)@β-CD from carbon, oxygen, iron, and cobalt with atomic % of 58.50, 38.61, 1.98, and 0.91%, respectively. The cobalt spectrum revealed the presence of three oxidation valences of cobalt; zero-valent cobalt since the characteristic peaks of Co^0^ 2p_3/2_ and Co^0^ 2p_1/2_ manifested at 781.59 and 798.30 eV^60,61^. The distinguishing peaks of the cobalt (II) species appeared at 785.33 and 803.40 eV ascribed to Co^2+^ 2p_3/2_ and Co^2+^ 2p_1/2_^62^. The XPS peaks of the cobalt (III) species; Co^3+^ 2p_3/2_ and Co^3+^ 2p_1/2_ illustrated at 788.83 and 808.39 eV^63^. Furthermore, the atomic percents of the Co^0^, Co^2+^, and Co^3+^ species in ZVCo-MIL-88 A(Fe)@β-CD were 30.64, 33.02, and 23.82%, respectively. The iron spectrum demonstrated the belonging peaks to the ferrous ions of 2p_3/2_ and 2p_1/2_ at the binding energy of 711.92 and 725.65 eV with a total atomic % of 55.76%. In addition, the related XPS peaks of the ferric species of 2p_3/2_ and 2p_1/2_ manifested at 714.82 and 729.89 eV with an atomic % of ferric ions of 22.77%^47^. The carbon spectrum elucidated three carbon functional groups; C-OH, C-O-C, and O-C = O at the binding energy of 284.99, 286.64, and 288.73 eV with atomic % of 42.16, 33.59, and 24.25%, sequentially^64^. The oxygen spectrum clarified the corresponding peaks of M-O, C = O, and O-H at 530.35, 531.65, and 532.76 eV with atomic % of 4.06, 15.5, and 80.44%, respectively^65^.

### Optimization of the cr(VI) adsorption

#### Identify the optimal ZVCo proportion

**Fig. 5:**
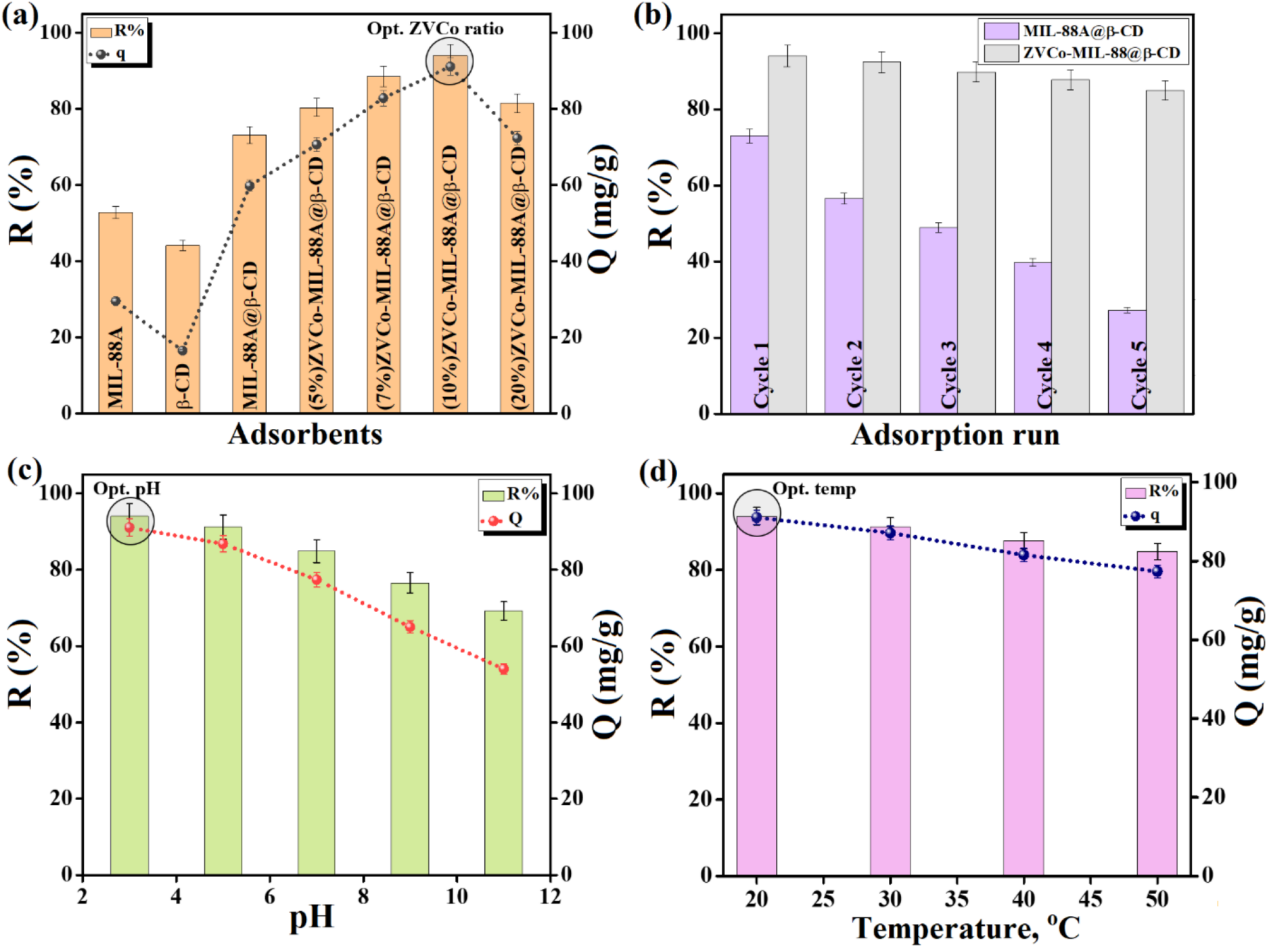
Experimental results of the Cr(VI) adsorption onto ZVCo-MIL-88 A(Fe)@β-CD; (**a**) Comparison study, (**b**) Recycling test of MIL-88 A(Fe)@β-CD and ZVCo-MIL-88 A(Fe)@β-CD for five Cr(VI) adsorption runs, (**c**) determine the optimal pH, and (**d**) investigate the thermodynamic state.

The adsorption behaviors of MIL-88 A(Fe), β-CD, and MIL-88 A(Fe)@β-CD toward adsorbing the Cr(VI) ions were examined at the same adsorption parameters, as depicted in Fig. 5a. The removal capabilities of MIL-88 A(Fe), β-CD, and MIL-88 A(Fe)@β-CD toward Cr(VI) were 52.78, 44.09, and 73.07%, while their adsorption capacities were 29.47, 16.49, and 59.77 mg/g, respectively. This experimental finding denoted the significance of supporting MIL-88 A(Fe) over the β-CD surface; however, the adsorption capacity of the MIL-88 A(Fe)@β-CD composite still required further improvements. In addition, the MIL-88 A(Fe)@β-CD separation by centrifugation was difficult, so the syringe filter was used as a secondary separation tool after centrifugation. This technique took a long time to separate MIL-88 A(Fe)@β-CD from the adsorption system, rendering the measurements inaccurate and wasting a part of the MIL-88 A(Fe)@β-CD dosage. Consequently, MIL-88 A(Fe)@β-CD was decorated with the magnetic ZVCo nanoparticles, showing an enhancement in the adsorption efficiency of the Cr(VI) ions from 70.61 to 91.07 mg/g and the removal % from 80.33 to 94.02% by raising the decorated proportion of ZVCo from 5 to 10 wt%. Nonetheless, the over-elevating of the ZVCo proportion to 20 wt% dwindled the removal % of Cr(VI) to 81.45% and the adsorption capacity to 72.29 mg/g, which could be anticipated by blocking the ZVCo-MIL-88 A(Fe)@β-CD pores by the excess proportion of ZVCo. Thus, 10 wt% of ZVCo is the optimal decorated proportion in the ZVCo-MIL-88 A(Fe)@β-CD composite.

On the other hand, a reusability test was performed on MIL-88 A(Fe)@β-CD and ZVCo-MIL-88 A(Fe)@β-CD to confirm the importance of the decorated ZVCo in boosting the recycling property of MIL-88 A(Fe)@β-CD (Fig. 5b). As expected, the MIL-88 A(Fe)@β-CD suffered a low recycling character since the removal % of Cr(VI) declined from 73.07 to 27.24% after five runs. This inferior recyclability of MIL-88 A(Fe)@β-CD because of its separation difficulty and mass loss during completing the separation by the syringe filter. Contrariwise, the magnetic character of ZVCo-MIL-88 A(Fe)@β-CD endowed it with fast and perfect separation, so the composite revealed an excellent recycling behavior during five Cr(VI) adsorption runs with a slight decrease in the removal % from 94.02 to 84.98%.

#### Identifying the optimal pH

The optimal pH to adsorb Cr(VI) on ZVCo-MIL-88 A(Fe)@β-CD was determined by studying the adsorption % of Cr(VI) at various acidic and alkaline media. The Cr(VI) ions are poly-protic species that have many forms at different pHs; Cr(VI) presents in acidic media (2–6) as Cr_2_O_7_^2−^/ HCrO_4_^−^, while at neutral and alkaline media (pH > 6), it exists in the CrO_4_^2−^ form^66^. As exhibited in Fig. 5c, the higher adsorption efficiency of Cr(VI) was recorded at pH = 3, where the R% and Q fulfilled 94.02% and 91.07 mg/g sequentially. Furthermore, a noticeable diminution of the adsorption aptitude of Cr(VI) was observed by augmenting the pH media over pH = 3, attaining R% = 69.24% and Q = 54.05 mg/g. Thereby, it was deduced the effectiveness of the electrostatic/repulsion forces on the Cr(VI) adsorption since at the highly acidic medium, the ZVCo-MIL-88 A(Fe)@β-CD carried high positive charges that could grasp the Cr(VI) ions onto its surface by electrostatic forces. On the contrary, in the alkaline and neutral media, the negative charges on the ZVCo-MIL-88 A(Fe)@β-CD surface played a role in inhibiting the adsorption process of Cr(VI) by repulsing the ions out of the composite via the electrostatic repulsion forces^67^. These experimental outcomes were typically agreed with the abovementioned zeta potential measurements, showing that the most favorable pH to adsorb Cr(VI) onto ZVCo-MIL-88 A(Fe)@β-CD was pH = 3.

#### Identifying the optimal adsorption temperature

The Cr(VI) removal efficiency by ZVCo-MIL-88 A(Fe)@β-CD was studied at different system temperatures, as demonstrated in Fig. 5d. The capacity of the Cr(VI) removal dwindled by elevating the temperature from room temperature to 55 °C, where R% and Q declined by 9.2% and 13.75 mg/g, respectively. Such a decrease in the removal efficacy of Cr(VI) by raising the adsorption temperature could be explained by: (i) the increase in the Brownian movement of the Cr(VI) ions at a higher temperature than the room temperature, hindering ions from reaching the ZVCo-MIL-88 A(Fe)@β-CD surface. (ii) the high temperature can raise the kinetic energy of the Cr(VI) ions, causing the desorption of the ions out of ZVCo-MIL-88 A(Fe)@β-CD.

#### Identifying the ideal dose of ZVCo-MIL-88 A(Fe)@β-CD

**Fig. 6:**
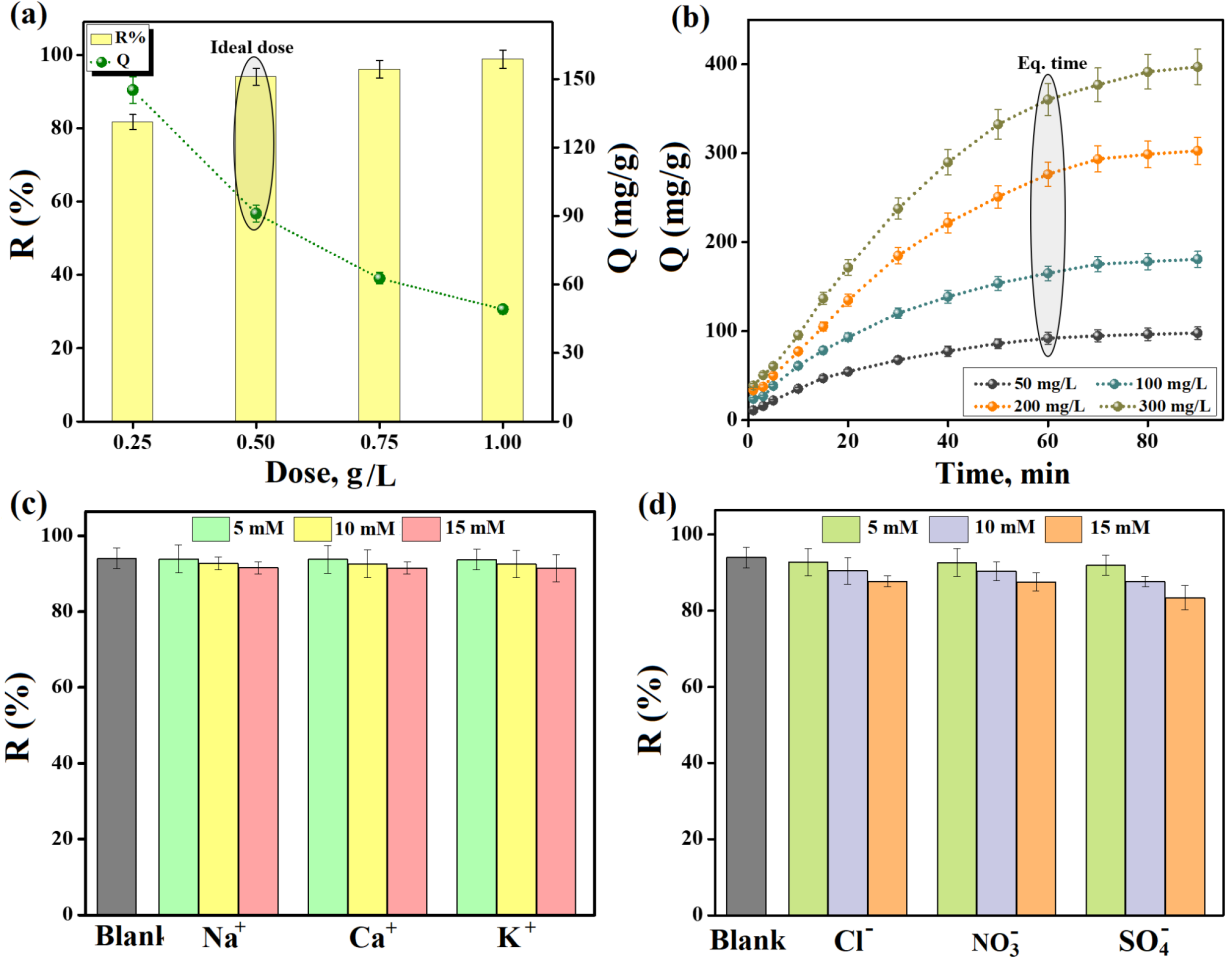
Experimental results of the Cr(VI) adsorption onto ZVCo-MIL-88 A(Fe)@β-CD; (**a**) identify the ideal dose, (**b**) evaluate the equilibrium time and (**c**,**d**) determine the influence of interfering ions.

Sequential adsorption experiments proceeded by different ZVCo-MIL-88 A(Fe)@β-CD doses to define the ideal dose to adsorb Cr(VI), as represented in Fig. 6a. The removal aptitude of Cr(VI) increased from 81.66 to 98.88% by elevating the mass of the added ZVCo-MIL-88 A(Fe)@β-CD in the adsorption system from 0.25 to 1.00 g/L, where the accessible adsorption sites get larger by high adsorbent mass. On the other hand, the capacity of the adsorbed Cr(VI) declined from 145.19 to 49.16 mg/g by this dose elevation, owing to the larger available binding active groups compared to the presented Cr(VI) ions in the adsorption system.

#### Identifying the equilibrium time at varies Cr(VI) concentrations

The equilibrium time of the adsorption process of Cr(VI) onto ZVCo-MIL-88 A(Fe)@β-CD was determined at varied Cr(VI) concentrations, as illustrated in Fig. 6b. The capacity of the Cr(VI) adsorption improved from 97.79 to 396.95 mg/g by increasing the Cr(VI) concentrations from 50 to 300 mg/L. Furthermore, the equilibrium time was recorded at 60 min, indicating the fast adsorption advantage of ZVCo-MIL-88 A(Fe)@β-CD. The removal aptitude decreased from 98.01 to 64.35% by augmenting the Cr(VI) concentrations because of the deficient active functional groups compared to high Cr(VI) anions Fig. S1.

#### Identifying the impact of interfering ions

The influence of the presented interfering anions and cation in the actual wastewater on the aptitude of the Cr(VI) adsorption onto the ZVCo-MIL-88 A(Fe)@β-CD adsorbent was studied and the experimental observations are illustrated in Fig. 6c,d. Interestingly, the existence of cations, such as calcium, sodium, and potassium species in the adsorption medium almost did not affect the capacity of the adsorbed Cr(VI) onto the ZVCo-MIL-88 A(Fe)@β-CD surface even by raising the concentration of cations to 30 mM. In addition, the presence of anions like chloride and nitrate slightly declines the adsorption % of the Cr(VI) ions, where they could form a weak outer-sphere complexation with ZVCo-MIL-88 A(Fe)@β-CD that slightly shields Cr(VI) to reach the adsorbent surface. While the sulfate ions have the ability to create a potent inner-sphere complexation with ZVCo-MIL-88 A(Fe)@β-CD that significantly retarded adsorbing Cr(VI) onto the adsorbent surface, decreasing the adsorption % of Cr(VI) by 10.53% when the sulfate concentration was 15 mM.

### Mechanistic study

#### Investigating the type of interaction of the cr(VI) adsorption

Kinetic and isotherm assessments were applied to identify the controlling interaction in the Cr(VI) adsorption reaction by ZVCo-MIL-88 A(Fe)@β-CD. So, the adsorption experimental data of Cr(VI) during 90 min were analyzed by kinetic models, comprising Pseudo first order, Pseudo second order, and Elovich (Equations S1-S3)^68^. In addition, the equilibrium data were inspected by isotherm models like Freundlich, Temkin, D-R, and Langmuir (Equations S4-S7)^69^.

**Fig. 7:**
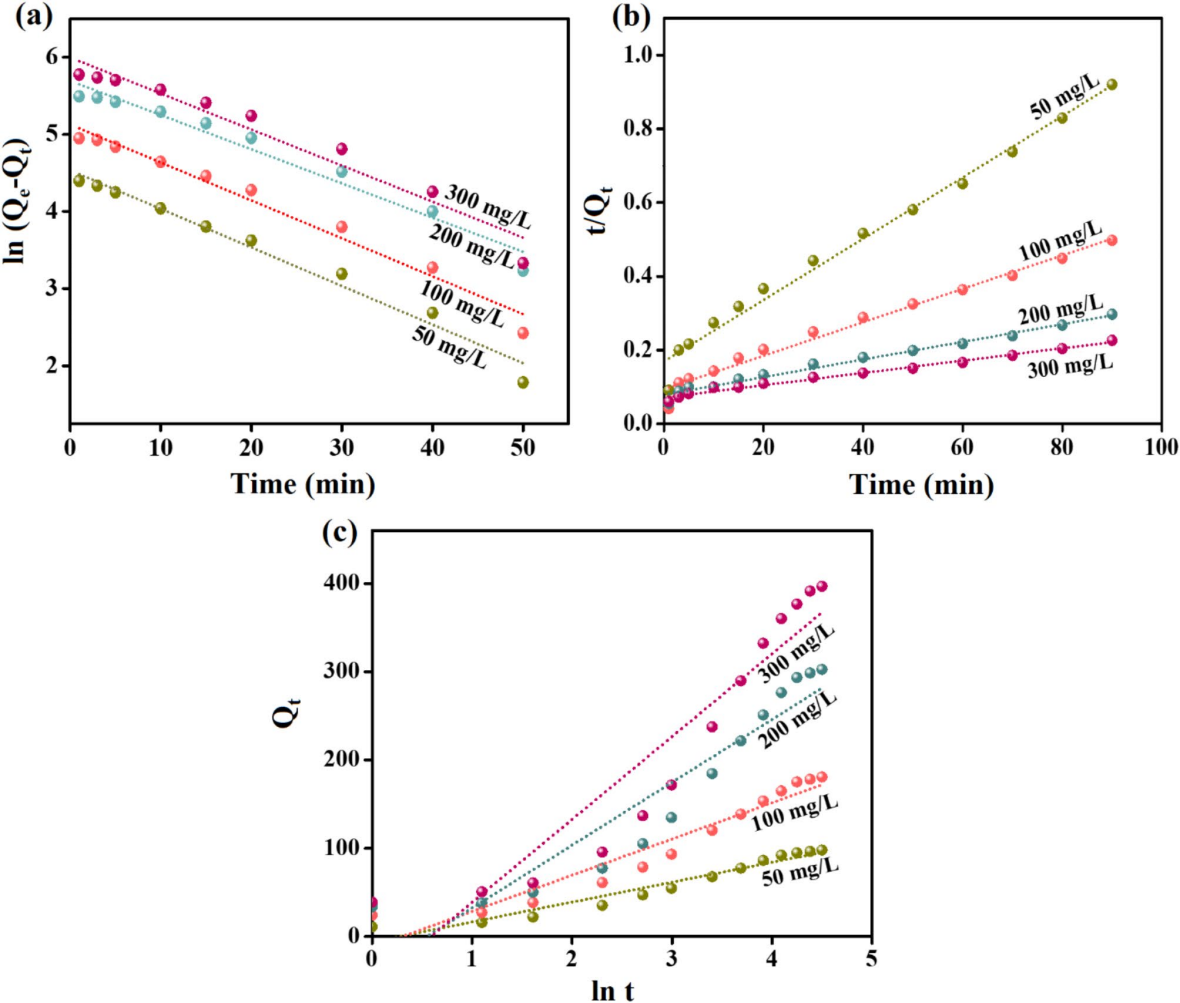
Experimental data analysis of adsorption of Cr(VI) onto ZVCo-MIL-88 A(Fe)@β-CD by (**a**) Pseudo first order, (**b**) Pseudo second order, and (**c**) Elovich.

The kinetics curves (Fig. 7a–c) implied that the Cr(VI) adsorption reaction proceeded by bonding the Cr(VI) ions with the active functional groups of ZVCo-MIL-88 A(Fe)@β-CD by chemical interactions since the Pseudo second order is the best kinetic model to represent the adsorption reaction. This result can be ascribed to the closeness between the calculated equilibrium adsorption capacity under Pseudo second order with the experimental data (Table 1). Furthermore, the correlation values of the Pseudo second-order curves are higher than those of the Pseudo first order. The Elovich model denoted the Cr(VI) adsorption favorability since the adsorption rates of the Cr(VI) anions toward the surface of ZVCo-MIL-88 A(Fe)@β-CD are greater than their desorption rates.

**Table 1:** Pseudo first order, Pseudo second order, and Elovich models parameters for adsorption of cr(VI) onto ZVCo-MIL-88 A(Fe)@β-CD.

Kinetic models and parameters	Concentration (mg/L)				
	50	100	200	300	
Q_e, Exp_ (mg/g)	92.06	164.96	276.34	360.31	
Pseudo first order					
Q_e, Cal_ (mg/g)	120.48	222.22	316.66	488.23	
k_1_ (min^− 1^)	0.050	0.049	0.044	0.046	
R^2^	0.978	0.976	0.966	0.951	
Pseudo second order					
Q_e, Cal_ (mg/g)	93.14	168.59	297.04	401.61	
k_2_ (g/mg min)	6.8 × 10^− 4^	3.7 × 10^− 4^	1.4 × 10^− 4^	8.6 × 10^− 4^	
R^2^	0.986	0.981	0.983	0.987	
Elovich					
α (mg/g min)	29.71	56.33	123.38	167.00	
β (g/mg)	4.4 × 10^− 2^	2.4 × 10^− 2^	1.4 × 10^− 2^	1.1 × 10^− 2^	
R^2^	0.945	0.924	0.887	0.887	

**Fig. 8:**
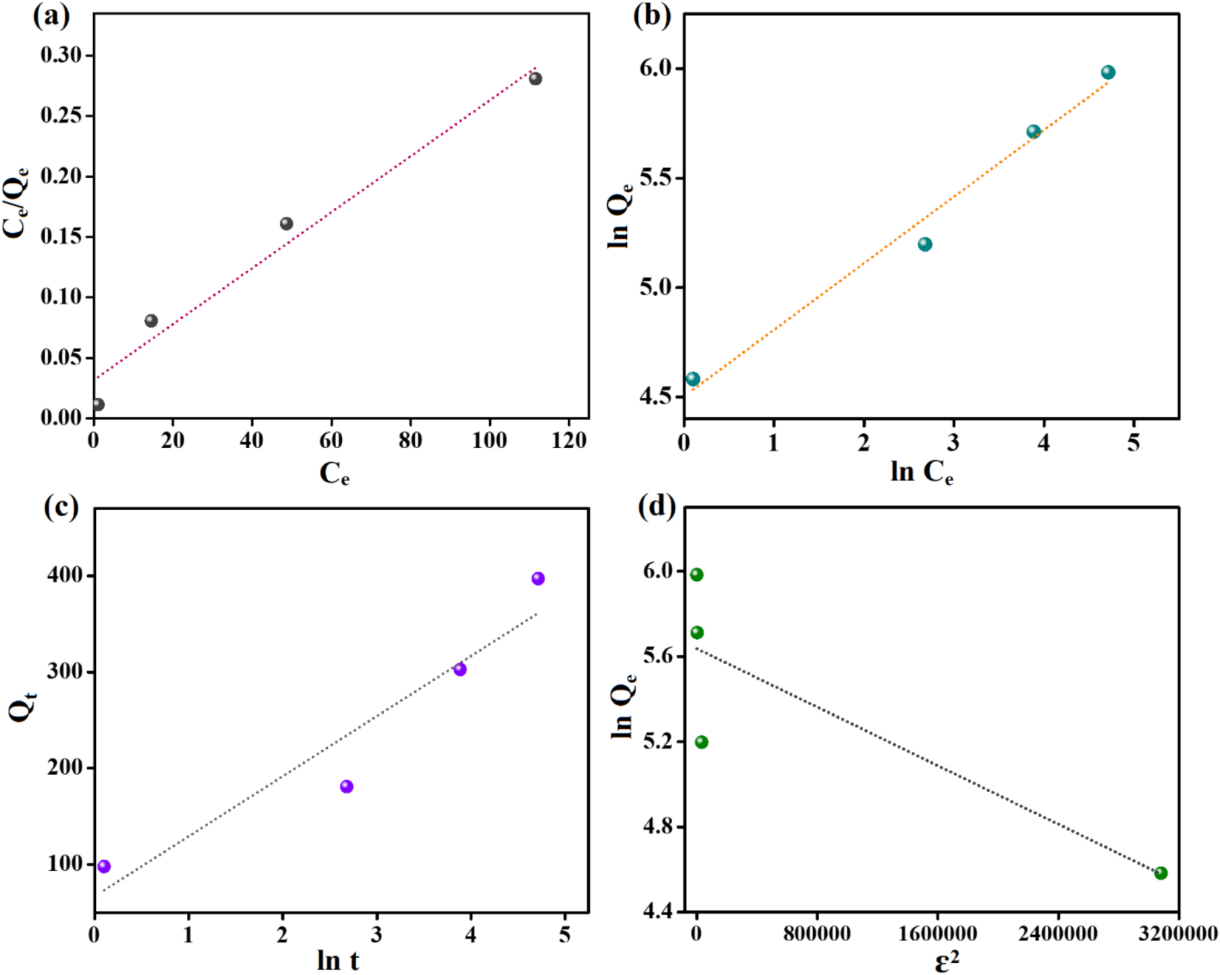
Experimental data analysis of adsorption of Cr(VI) onto ZVCo-MIL-88 A(Fe)@β-CD by (**a**) Langmuir, (**b**) Freundlich, (**c**) Temkin, and (**d**) D-R.

The isotherms curves (Fig. 8a–d) clarified that the physical and chemical interaction dominated the Cr(VI) adsorption reaction onto ZVCo-MIL-88 A(Fe)@β-CD, where the correlation value of Freundlich and Langmuir are almost the same, as elucidated in Table 2. The computed highest adsorption aptitude of ZVCo-MIL-88 A(Fe)@β-CD toward the Cr(VI) anions was 434.78 mg/g at 25 °C. The n value of Freundlich confirmed the suitability of the ZVCo-MIL-88 A(Fe)@β-CD surface to adsorb the Cr(VI) anions, where n was about 3.29. The b value of Temkin indicated the domination of the physical interaction between Cr(VI) and ZVCo-MIL-88 A(Fe)@β-CD.

**Table 2:** Parameters of Langmuir, Freundlich, Temkin, and D-R isotherms for the adsorption cr(VI) onto ZVCo-MIL-88 A(Fe)@β-CD.

Isotherm model	Parameters	Value
Langmuir	Q_max_ (mg/g) K_L_ (L/mg) R^2^	434.78 0.0728 0.972
Freundlich	n K_F_ ((mg/g) (L/mg)^1/n^) R^2^	3.29 90.29 0.983
Temkin	k_T_ (L/mg) b_T_ (KJ/mol) R^2^	2.90 39.63 0.909
D-R	Q_s_ (mg/g) K_DR_ (mol^2^/J^2^) E (kJ/mol) R^2^	280.62 3 × 10^− 7^ 1.291 0.729

#### Identify the thermodynamics of the cr(VI) adsorption

Thermodynamics of the adsorption reaction of Cr(VI) onto ZVCo-MIL-88 A(Fe)@β-CD was elucidated by determining the thermodynamic parameters, comprising change in Gibbs free energy (∆G°), change in enthalpy (∆H°), and change in entropy (∆S°)^70^. The linear Van’t Hoff’s curve (Eqs. 3, 4) identified ∆H° and ∆S° from the slope and intercept, respectively, and then ∆G° was calculated from Eq. (5). Table 3 clarified the resultant thermodynamic parameters of Cr(VI) onto ZVCo-MIL-88 A(Fe)@β-CD.

3 $$\:=exp\left[\frac{\varDelta\:{S}^{^\circ\:}}{R}-\left(\frac{\varDelta\:H}{R}\right).\:\frac{1}{\:T}\right]\:$$

4 $$\:{K}_{d}=\frac{{C}_{Ae}}{{C}_{o}}$$

5 $$\Delta G^{0} = \Delta H^{0} - T\Delta S^{0}$$

Where, K_d_ (L/g) is the distribution constant, C_Ae_ is the concentration of the adsorbed Cr(VI) onto ZVCo-MIL-88 A(Fe)@β-CD, C_o_ is the initial concentration of Cr(VI), R (J/mol K) is the gas constant, and T (K) is the absolute temperature.

**Table 3:** Thermodynamic parameters of the adsorption of cr(VI) onto ZVCo-MIL-88 A(Fe)@β-CD composite.

T (K)	Thermodynamic parameters		
	∆G° (kJ/mol)	∆H° (kJ/mol)	∆S° (J/mol.K)
293 303 313 323	2.25 2.33 2.41 2.48	-2.59	-7.70

The thermodynamics investigation demonstrated the positive magnitude of ∆G°, which suggested the unspontaneity of the adsorption of the Cr(VI) ions onto ZVCo-MIL-88 A(Fe)@β-CD composite. In addition, the negative magnitude of ∆H° elucidates the exothermic character of the adsorption Cr(VI)/ZVCo-MIL-88 A(Fe)@β-CD system, and the negative ∆S° magnitude implies the ordered solid-solution interface^71^.

#### Investigating the interactions inside the adsorption system

**Fig. 9:**
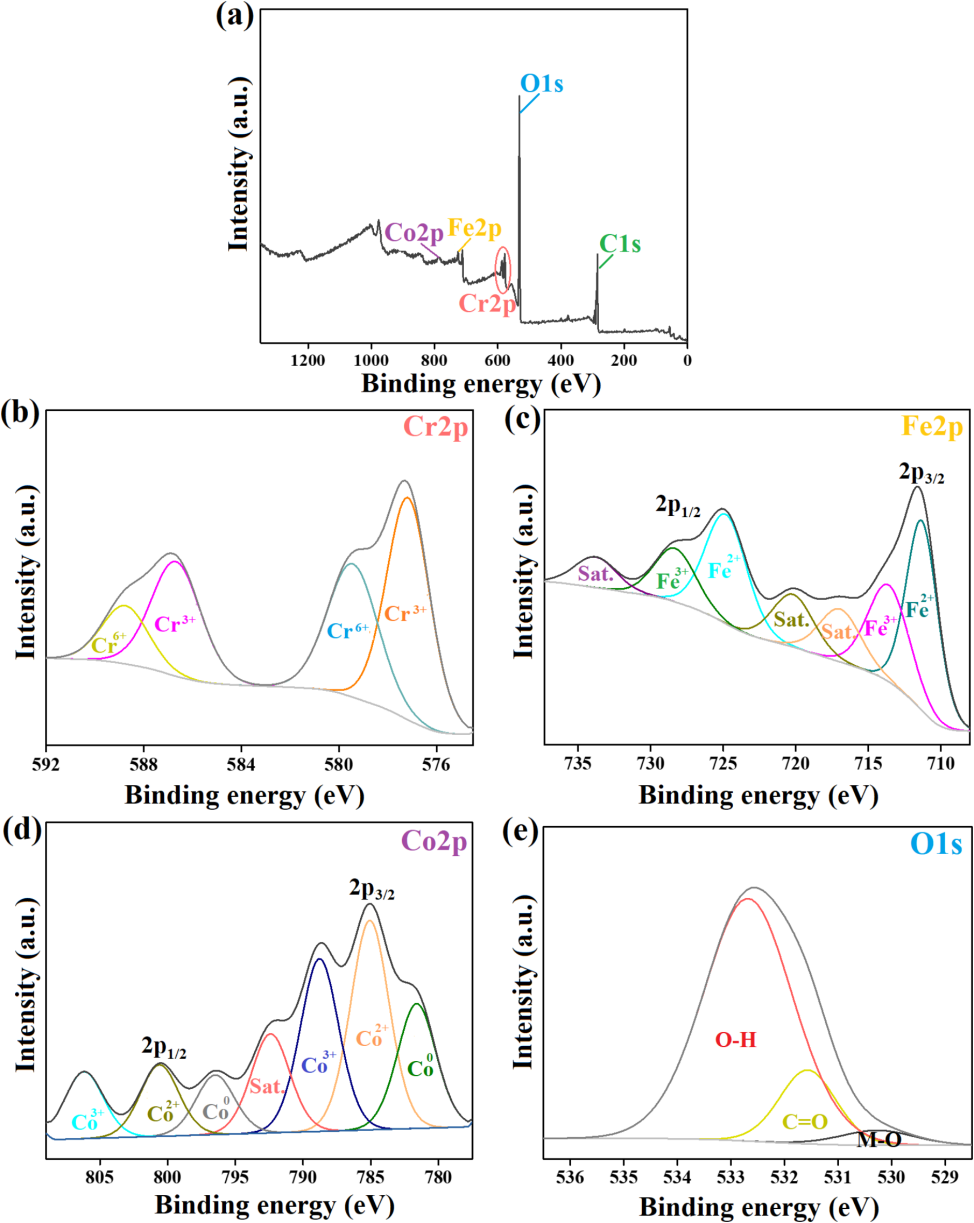
The XPS spectra of the Cr(VI)-loaded ZVCo-MIL-88 A(Fe)@β-CD; (**a**) Wide-spectrum, (**b**) Cr2p, (**c**) Fe2p, (**d**) Co2p, and (**e**) O1s.

According to the kinetic and isotherm assessments, physical and chemical interactions control the adsorption of Cr(VI) onto ZVCo-MIL-88 A(Fe)@β-CD. XPS of the pure and used ZVCo-MIL-88 A(Fe)@β-CD suggested how the Cr(VI) interacted with the functional groups of the ZVCo-MIL-88 A(Fe)@β-CD composite. The wide-scan spectrum of the used ZVCo-MIL-88 A(Fe)@β-CD (Fig. 9a) clarified the distinguishing peak of Cr 2p at 578.29 eV, confirming the Cr(VI) adsorption onto the composite.

##### Chemical interactions

*Reduction reaction*

In light of previous studies regarding Cr(VI) adsorption, it was supposed that reducing Cr(VI) to Cr(III) is a dominant adsorption pathway. Notably, the Cr 2p spectrum (Fig. 9b) demonstrated the belonging peaks to Cr(VI) at 579.43 and 589.57 eV, while the Cr(III) peaks manifested at 577.16 and 587.13 eV, implying the participation of the reduction reaction in the Cr(VI) adsorption process (Eqs. 6, 7). Furthermore, the atomic % of Cr(VI) and Cr(III) were 29.12 and 70.88%, which denoted that the reduction reaction controlled the highest capacity of the adsorbed Cr(VI) onto ZVCo-MIL-88 A(Fe)@β-CD. The ability of ZVCo in situ to produce Co^2+^ facilitates its participation in the adsorption mechanism via the reduction reaction^28^, as elucidated in Eq. (8). Furthermore, the available unsaturated iron species can contribute to the Cr(VI) reduction reaction, as represented in Eq. (9). The redox potential of Fe^3+^/Fe^2+^ and Co^3+^/Co^2+^ are 0.77 and 1.808 V, respectively, which enables the Fe^2+^ species to recover the Co^3+^ species (Eq. 10). The shifting in the Fe 2p and Co 2p peaks reflected their participation in adsorbing Cr(VI) onto the ZVCo-MIL-88 A(Fe)@β-CD surface (Fig. 9c,d).

6 $${\text{HCrO}}_{4}^{ - } + {\text{ }}7{\text{H}}^{ + } + {\text{ }}3{\text{e}} \to {\text{Cr}}^{{3 + }} + 4{\text{H}}_{2} {\text{O}}$$

7 $${\text{Cr}}_{2} {\text{O}}_{7}^{{2 - }} + {\text{ }}14{\text{H}}^{ + } + {\text{ }}6{\text{ e}} \to 2{\text{ Cr}}^{{3 + }} + {\text{ }}7{\text{ H}}_{2} {\text{O}}$$

8 $$3{\text{ Co}}^{{2 + }} + {\text{Cr}}^{{6 + }} \to 3{\text{ Co}}^{{3 + }} + {\text{Cr}}^{{3 + }}$$

9 $$3{\text{ Fe}}^{{2 + }} + {\text{Cr}}^{{6 + }} \to 3{\text{ Fe}}^{{3 + }} + {\text{Cr}}^{{3 + }}$$

10 $${\text{Fe}}^{{2 + }} + {\text{Co}}^{{3 + }} \to {\text{Fe}}^{{3 + }} + {\text{Co}}^{{2 + }}$$

*Coordination bonds*

The coordination bond is a vital adsorption pathway during removing Cr(VI) from wastewater, where the electron donor groups (EDG)-containing adsorbent could bond with the species by a coordination bond, forming a complex. Interestingly, the ZVCo-MIL-88 A(Fe)@β-CD composite contains more than one EDG, which are hydroxyl and carboxyl that could interact with the Cr(VI) ions by a coordination bond to form a complex. The oxygen spectrum of the used ZVCo-MIL-88 A(Fe)@β-CD exhibited a slight change in the position of the peaks compared with the spectrum of the neat composite, which could be explained by the contribution of the oxygenated functional groups in adsorbing Cr(VI) from their bulk solution (Fig. 9e). In addition, a coordination bond could be formed between the reduced chromium ions onto the composite surface, which avoids escaping Cr(III) ions to purified water^72^.

##### Physical interactions

*Electrostatic interaction*

Undoubtedly, electrostatic interaction is the most effective adsorption pathway that is regarded during the setup of the adsorption system since it is essential to select adsorbent with opposite surface charge to the targeted adsorbate. Noteworthy, pioneering studies have reported that the electrostatic interaction is the initial step that grasps the pollutant from the bulk solution to the adsorbent surface, then the adsorbed pollutant could interact with the adsorbent by other interactions, such as reduction, coordination bond, etc. The ZVCo-MIL-88 A(Fe)@β-CD composite possesses a high positive surface charge fulfilled 21.11 mV at pH = 3, which magically attracts the anionic CrO_4_^2−^ from wastewater to its surface by the potent electrostatic interactions.

*Pore filling*

Porosity is one of the significant criteria of efficient adsorbents since the pores can uptake the pollutants inside them, with one condition, the pore size of the adsorbent be larger than the radius of pollutants. Interestingly, the ZVCo-MIL-88 A(Fe)@β-CD composite contains loose cavities of β-CD that participate in the adsorption processes throughout the pore-filling mechanism. The pore radius of Cr(VI) is 0.052 nm and the cavity diameter of β-CD ranges from 0.6 to 0.65 nm, reflecting the breadth of the β-CD cavities to contain amounts of the Cr(VI) ions.

**Fig. 10:**
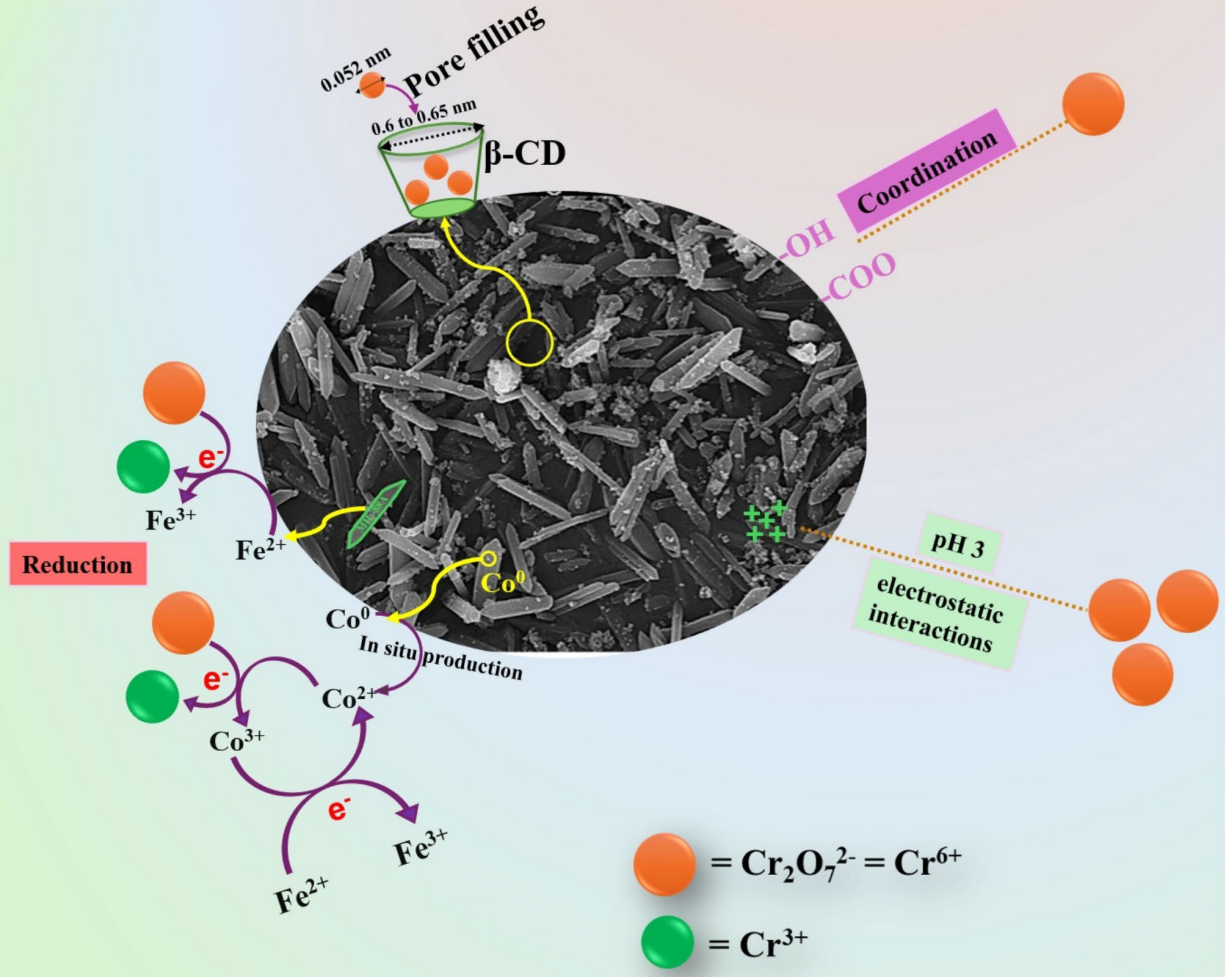
Schematic representation of the Cr(VI) adsorption onto the ZVCo-MIL-88 A(Fe)@β-CD composite.

Accordingly, the Cr(VI) species adsorbed on the ZVCo-MIL-88 A(Fe)@β-CD composite throughout the chemical pathways, which are reduction interaction and coordination bonds; in addition, the physical pathways, including electrostatic interaction and pore filling, as shown in Fig. 10.

##### Comparison study

Table 4 lists a comparison between our study and previous pioneering research work, taking into consideration clarifying the optimum adsorption condition and adsorption mechanism. In light of this compiled data, our study provides an efficient adsorbent of ZVCo-MIL-88 A(Fe)@β-CD for removing the detrimental Cr(VI) with high efficacy in a short processing time.

**Table 4:** A comparison study between the adsorption performance of ZVCo-MIL-88 A(Fe)@β-CD and other previous reported adsorbents.

Adsorbent	q_max_ (mg/g)	Eq. time (min)	Opt. pH	Adsorption mechanism	Refs.
Activated TETA-PP	312.50	30	1.6	Reduction reaction, surface complexation, and electrostatic interaction	^73^
CdS/PEI-BC/Cs	513.43	30	3	Electrostatic interaction, reduction reaction, and complexation	^74^
ZnBDC/C-Cs	255	25	5	Electrostatic interaction and weak cation-π interaction	^75^
WBT	9.14	45	4	Electrostatic interaction and reduction reaction	^76^
FeYBC	24.37	120	2.99–6.07	Complexation and reduction reaction	^77^
ATP–CPBr@CA	302.11	120	2	Electrostatic interaction, ion-exchange, inner-/outer-complexation, reduction, pore-filling, and coordination bonds	^78^
PBN	384.62	180	1.5	Electrostatic interaction	^79^
MPA-10	538.97	200	2	Electrostatic interaction, reduction reaction, and chelation interaction	^80^
GO-NH_2_@CA-NH_2_	410.21	60	2	Electrostatic interaction, reduction reaction, and coordinate–covalent bond	^72^
Cs-stabilized FeS	119.00	120	3	Electrostatic interaction and reduction reaction	^81^
ZVCo-MIL-88 A(Fe)@β-CD	434.78	60	3	Pore-filling, electrostatic interaction, reduction interaction, and coordination bond	This study

## Conclusion

Shortly, the magnetic adsorbent of the ZVCo-MIL-88 A(Fe)@β-CD composite was synthesized by the post-synthetic procedure. The VSM plot represented a super-paramagnetic hysteresis loop of ZVCo-MIL-88 A(Fe)@β-CD with a higher saturation magnetization of 17.19 emu/g. Moreover, the zeta potential curve clarified that the zero-charge point of the composite surface was at pH = 7.23, with a zeta potential value of 21.11 mV at pH = 3. The lab experiments demonstrated the eminent adsorbability and reusability of ZVCo that boosted the Cr(VI) removal % and reusability of MIL-88 A(Fe)@β-CD from 73.07 to 94.02% and 27.24 and 84.98% after its decoration with 10 wt% of ZVCo. Notably, the best adsorption performance of ZVCo-MIL-88 A (Fe)@β-CD towards the Cr(VI) ions occurred at a highly acidic medium and room temperature after an equilibrium time of an hour. The data analysis of the experimental results of the Cr(VI) adsorption implied the preference of the Freundlich and Langmuir isotherm models and Pseudo second-order kinetic model to model the adsorption process inside the Cr(VI)/ZVCo-MIL-88 A(Fe)@β-CD adsorption system. The mechanistic investigation supposed that reduction reaction, coordination bonds, electrostatic interactions, and pore-filling are the possible pathways to adsorb Cr(VI) onto ZVCo-MIL-88 A(Fe)@β-CD.

## Electronic supplementary material

Below is the link to the electronic supplementary material.

Supplementary Material 1

## Data Availability

The datasets used and/or analysed during the current study available from the corresponding author on reasonable request.

## References

[1] Ileperuma, O. *Environmental pollution in Sri Lanka: a review.* (2000).

[2] Chattopadhyay, T. et al. Insights into the selective anticancer activity of [SnIV (L)(cl) 2 (OH2)][L=(E)-N, N-diethyl-2-(2-hydroxy-3-methoxybenzylidene) hydrazinecarbothioamide] at thiosemicarbazone appended tin (IV) site. *Inorg. Chem. Commun.* **165**, 112525 (2024).

[3] Chen, X. et al. Nano-encapsulation of Epigallocatechin Gallate (EGCG) within Zeolitic Imidazolate Framework-8 (ZIF-8) and Controlled Release of EGCG (表没食子儿茶素没食子酸酯 (Egcg) 在沸石咪唑酯骨架-8 (Zif-8) 中的纳米封装以及 Egcg 的控制释放). Available at SSRN 5000837.

[4] Eleryan, A. et al. Isothermal and kinetic screening of methyl red and methyl orange dyes adsorption from water by Delonix regia biochar-sulfur oxide (DRB-SO). *Sci. Rep.* **14**(1), 13585 (2024). 38866857 10.1038/s41598-024-63510-0 PMC11169550

[5] Nazir, M. A. et al. Heterointerface engineering of water stable ZIF-8@ ZIF-67: adsorption of rhodamine B from water. *Surf. Interfaces*. **34**, 102324 (2022).

[6] Ukhurebor, K. E. et al. Effect of hexavalent chromium on the environment and removal techniques: a review. *J. Environ. Manage.* **280**, 111809 (2021). 33360556 10.1016/j.jenvman.2020.111809

[7] Sharma, A. et al. Chromium bioaccumulation and its impacts on plants: an overview. *Plants* **9**(1), 100 (2020). 31941115 10.3390/plants9010100 PMC7020214

[8] Madhusudan, P., Lee, C. & Kim, J. O. Synthesis of Al2O3@ Fe2O3 core–shell nanorods and its potential for fast phosphate recovery and adsorption of chromium (VI) ions from contaminated wastewater. *Sep. Purif. Technol.* **326**, 124691 (2023).

[9] Kumaraguru, K. et al. A systematic analysis of hexavalent chromium adsorption and elimination from aqueous environment using brown marine algae (Turbinaria ornata). *Biomass Convers. Biorefin.* **13**(9), 8223–8238 (2023).

[10] Omer, A. M. et al. Construction of efficient Ni-FeLDH@ MWCNT@ cellulose acetate floatable microbeads for cr (VI) removal: performance and mechanism. *Carbohydr. Polym.* **311**, 120771 (2023). 37028881 10.1016/j.carbpol.2023.120771

[11] Eltaweil, A. S. et al. *Magnetic Hierarchical flower-like Fe3O4@ ZIF-67/CuNiMn-LDH Catalyst with Enhanced Redox Cycle for Fenton-like Degradation of Congo Red: Optimization and Mechanism* 1–17 (Environmental Science and Pollution Research, 2023). 10.1007/s11356-023-27430-2 PMC10293427 37219772

[12] Liu, Y. et al. Ferrous disulfide and iron nitride sites on hydrochar to enhance synergistic adsorption and reduction of hexavalent chromium. *Bioresour. Technol.* **388**, 129770 (2023). 37714497 10.1016/j.biortech.2023.129770

[13] Wu, C. et al. Reduction and precipitation of chromium (VI) using a palladized membrane biofilm reactor. *Water Res.* **249**, 120878 (2024). 38007896 10.1016/j.watres.2023.120878

[14] Liu, B. et al. Removal of Chromium species by Adsorption: Fundamental principles, newly developed adsorbents and future perspectives. *Molecules* **28**(2), 639 (2023). 36677697 10.3390/molecules28020639 PMC9861687

[15] Garg, R. et al. Rapid adsorptive removal of chromium from wastewater using walnut-derived biosorbents. *Sci. Rep.* **13**(1), 6859 (2023). 37100812 10.1038/s41598-023-33843-3 PMC10133242

[16] Eltaweil, A. S. et al. Fabrication of UiO-66/MIL-101 (fe) binary MOF/carboxylated-GO composite for adsorptive removal of methylene blue dye from aqueous solutions. *RSC Adv.* **10**(32), 19008–19019 (2020). 35518294 10.1039/d0ra02424d PMC9053870

[17] Abd El-Monaem, E. M. et al. *Adsorption of Nitrophenol onto a Novel Fe3O4-κ-carrageenan/MIL-125 (Ti) Composite: Process Optimization, Isotherms, Kinetics, and Mechanism* 49301–49313 (Environmental Science and Pollution Research, 2023). 17. 10.1007/s11356-023-25678-2 PMC10104928 36773266

[18] Howarth, A. J. et al. Chemical, thermal and mechanical stabilities of metal–organic frameworks. *Nat. Rev. Mater.* **1**(3), 1–15 (2016).

[19] Peters, A. W. et al. Toward inexpensive photocatalytic hydrogen evolution: a nickel sulfide catalyst supported on a high-stability metal–organic framework. *ACS Appl. Mater. Interfaces*. **8**(32), 20675–20681 (2016). 27487409 10.1021/acsami.6b04729

[20] Wu, H. et al. Arsenic removal from water by metal-organic framework MIL-88A microrods. *Environ. Sci. Pollut. Res.* **25**, 27196–27202 (2018). 10.1007/s11356-018-2751-2 30027376

[21] Abd El-Monaem, E. M. et al. Enhanced Redox Cycle of Rod-shaped MIL-88A/SnFe2O4@ MXene sheets for Fenton-like degradation of Congo Red: optimization and mechanism. *Nanomaterials* **14**(1), 54 (2023). 38202509 10.3390/nano14010054 PMC10780543

[22] Liao, X. et al. Synthesis of (100) surface oriented MIL-88A-Fe with rod-like structure and its enhanced fenton-like performance for phenol removal. *Appl. Catal. B*. **259**, 118064 (2019).

[23] Lin, K. Y. A., Chang, H. A. & Hsu, C. J. Iron-based metal organic framework, MIL-88A, as a heterogeneous persulfate catalyst for decolorization of rhodamine B in water. *RSC Adv.* **5**(41), 32520–32530 (2015).

[24] Omer, A. M. et al. Fabrication of easy separable and reusable MIL-125 (Ti)/MIL-53 (fe) binary MOF/CNT/Alginate composite microbeads for tetracycline removal from water bodies. *Sci. Rep.* **11**(1), 23818 (2021). 34893701 10.1038/s41598-021-03428-z PMC8664953

[25] Hassani, A. et al. *Acetaminophen Removal from Aqueous Solutions through Peroxymonosulfate Activation by CoFe2O4/mpg-C3N4 Nanocomposite: Insight into the Performance and Degradation Kinetics* 101127 (Environmental Technology & Innovation, 2020).

[26] Elkady, M. et al. New insights into the activity of green supported nanoscale zero-valent iron composites for enhanced acid blue-25 dye synergistic decolorization from aqueous medium. *J. Mol. Liq.* **294**, 111628 (2019).

[27] Zhai, Y. et al. Highly efficient cobalt nanoparticles anchored porous N-doped carbon nanosheets electrocatalysts for Li-O2 batteries. *J. Catal.* **377**, 534–542 (2019).

[28] Zhou, G. et al. Efficient degradation of sulfamethoxazole using peracetic acid activated by zero-valent cobalt. *J. Environ. Chem. Eng.* **10**(3), 107783 (2022).

[29] Liu, J. Y., Zhang, X. & TIAN, B. Selective modifications at the different positions of cyclodextrins: a review of strategies. *Turk. J. Chem.* **44**(2), 261–278 (2020). 33488156 10.3906/kim-1910-43 PMC7671212

[30] Kurkov, S. V. & Loftsson, T. Cyclodextrins. *Int. J. Pharm.* **453**(1), 167–180 (2013). 22771733 10.1016/j.ijpharm.2012.06.055

[31] Ding, L. et al. Studies on a novel modified β-cyclodextrin inclusion complex. *J. Mol. Struct.* **979**(1–3), 122–127 (2010).

[32] Wankar, J. et al. Recent advances in host–guest self-assembled cyclodextrin carriers: implications for responsive drug delivery and biomedical engineering. *Adv. Funct. Mater.* **30**(44), 1909049 (2020).

[33] Wang, R. Q., Wei, X. B. & Feng, Y. Q. β-Cyclodextrin covalent organic framework for selective molecular adsorption. *Chemistry–A Eur. J.* **24**(43), 10979–10983 (2018). 10.1002/chem.201802564 29873120

[34] Tian, B., Liu, Y. & Liu, J. Smart stimuli-responsive drug delivery systems based on cyclodextrin: a review. *Carbohydr. Polym.* **251**, 116871 (2021). 33142550 10.1016/j.carbpol.2020.116871

[35] Jacob, S. & Nair, A. B. Cyclodextrin complexes: perspective from drug delivery and formulation. *Drug Dev. Res.* **79**(5), 201–217 (2018). 30188584 10.1002/ddr.21452

[36] Rajkumar, T. et al. Cyclodextrin-metal–organic framework (CD-MOF): from synthesis to applications. *J. Ind. Eng. Chem.* **72**, 50–66 (2019).

[37] Liu, Y. et al. β-Cyclodextrin-based hollow nanoparticles with excellent adsorption performance towards organic and inorganic pollutants. *Nanoscale* **11**(40), 18653–18661 (2019). 31584597 10.1039/c9nr07342f

[38] Fu, M. et al. Adsorption performance and mechanism of pectin modified with β-cyclodextrin for Zn2 + and Cu2+. *Int. J. Biol. Macromol.* **274**, 133563 (2024). 38950803 10.1016/j.ijbiomac.2024.133563

[39] Kekes, T., Kolliopoulos, G. & Tzia, C. Hexavalent chromium adsorption onto crosslinked chitosan and chitosan/β-cyclodextrin beads: novel materials for water decontamination. *J. Environ. Chem. Eng.* **9**(4), 105581 (2021).

[40] Wang, G. et al. Adsorption of dichromate ions from aqueous solution onto magnetic graphene oxide modified by β-cyclodextrin. *Environ. Sci. Pollut. Res.* **27**(24), 30778–30788 (2020). 10.1007/s11356-020-09389-6 32474786

[41] Wang, P. et al. Simultaneous removal of organic micropollutants and metals from water by a multifunctional β-cyclodextrin polymer-supported-polyaniline composite. *Chem. Eng. J.* **482**, 148826 (2024).

[42] Hammad, E. N. et al. Enhanced cr (VI) removal via CPBr-modified MIL-88A@ amine-functionalized GO: synthesis, performance, and mechanism. *Environ. Sci. Pollut. Res.* **31**(35), 47851–47865 (2024). 10.1007/s11356-024-33859-w 39009817

[43] Kakavandi, B. et al. Enhanced chromium (VI) removal using activated carbon modified by zero valent iron and silver bimetallic nanoparticles. *J. Environ. Health Sci. Eng.* **12**, 1–10 (2014). 25184050 10.1186/s40201-014-0115-5 PMC4147180

[44] Qiu, Y. et al. Removal mechanisms of cr (VI) and cr (III) by biochar supported nanosized zero-valent iron: synergy of adsorption, reduction and transformation. *Environ. Pollut.* **265**, 115018 (2020). 32806451 10.1016/j.envpol.2020.115018

[45] El-Monaem, E. M. A. et al. Cobalt nanoparticles supported on reduced amine-functionalized graphene oxide for catalytic reduction of nitroanilines and organic dyes. *Nano* **16**(04), 2150039 (2021).

[46] Liu, N. et al. Ultrathin graphene oxide encapsulated in uniform MIL-88A (Fe) for enhanced visible light-driven photodegradation of RhB. *Appl. Catal. B*. **221**, 119–128 (2018).

[47] Eltaweil, A. S. et al. Designing of SrFe2O4-decorated sulfur-MXene for super-fast adsorption of mercury. *J. Mol. Liq.* 125275 (2024).

[48] Omer, A. M. et al. Sustainable synthesis of magnetic petroleum coke/nonanyl chitosan composite for efficient removal of o-nitrophenol. *Sci. Rep.* **14**(1), 14463 (2024). 38914588 10.1038/s41598-024-64117-1 PMC11196280

[49] Li, N. et al. Construction of Z-scheme CuBi2O4/MIL-88A (Fe) heterojunctions with enhanced LED light driven photocatalytic cr (VI) reduction and antibacterial performance. *Appl. Surf. Sci.* **614**, 156249 (2023).

[50] Akgün, D. & Dükkancı, M. g-C3N4 supported Ag/AgCl@ MIL-88A MOF based triple composites for highly efficient diuron photodegradation under visible LED light irradiation. *J. Water Process Eng.* **51**, 103469 (2023).

[51] Yuan, C., Liu, B. & Liu, H. Characterization of hydroxypropyl-β-cyclodextrins with different substitution patterns via FTIR, GC–MS, and TG–DTA. *Carbohydr. Polym.* **118**, 36–40 (2015). 25542104 10.1016/j.carbpol.2014.10.070

[52] Markova-Deneva, I. Infrared spectroscopy investigation of metallic nanoparticles based on copper, cobalt, and nickel synthesized through borohydride reduction method. *J. Univ. Chem. Technol. Metall.* **45**(4), 351–378 (2010).

[53] Li, M. et al. Citric acid-modified MIL-88A(Fe) for enhanced photo-Fenton oxidation in water decontamination. *Sep. Purif. Technol.* **308**, 122945 (2023).

[54] Liu, C. et al. Photo-induced heterogeneous regeneration of Fe(II) in Fenton reaction for efficient polycyclic antibiotics removal and in-depth charge transfer mechanism. *J. Colloid Interface Sci.* **638**, 768–777 (2023). 36780855 10.1016/j.jcis.2023.02.010

[55] Song, W. et al. Cyclodextrin-erythromycin complexes as a drug delivery device for orthopedic application. *Int. J. Nanomed.* 3173–3186 (2011). 10.2147/IJN.S23530 PMC3252670 22228990

[56] Sadaquat, H. & Akhtar, M. Comparative effects of β-cyclodextrin, HP-β-cyclodextrin and SBE 7-β-cyclodextrin on the solubility and dissolution of docetaxel via inclusion complexation. *J. Incl. Phenom. Macrocycl. Chem.* **96**, 333–351 (2020).

[57] Hao, J. et al. Natural-product-tailored polyurethane: size-dictated construction of polypseudorotaxanes with cyclodextrin–triterpenoid pairs. *ACS Macro Lett.* **7**(9), 1131–1137 (2018). 35632944 10.1021/acsmacrolett.8b00560

[58] Manjunatha, M. et al. Determination of phase composition of cobalt nanoparticles using 59 Co internal field nuclear magnetic resonance. *J. Supercond. Novel Magn.* **32**, 3201–3209 (2019).

[59] Lu, A. H., Salabas, E. L. & Schüth, F. Magnetic nanoparticles: synthesis, protection, functionalization, and application. *Angew. Chem. Int. Ed.* **46**(8), 1222–1244 (2007). 10.1002/anie.200602866 17278160

[60] Vickers, S. M. et al. Mesoporous Mn-and La-doped cerium oxide/cobalt oxide mixed metal catalysts for methane oxidation. *ACS Appl. Mater. Interfaces* **7**(21), 11460–11466 (2015). 26000732 10.1021/acsami.5b02367

[61] Cai, N. et al. Meso-microporous carbon nanofibers with in-situ embedded Co nanoparticles for catalytic oxidization of azo dyes. *J. Mol. Liq.* **289**, 111060 (2019).

[62] Penke, Y. K. et al. Aluminum substituted cobalt ferrite (Co – Al – Fe) nano adsorbent for arsenic adsorption in aqueous systems and detailed redox behavior study with XPS. *ACS Appl. Mater. Interfaces* **9**(13), 11587–11598 (2017). 28257174 10.1021/acsami.6b16414

[63] Zhao, L. et al. Electro-oxidation of ascorbic acid by cobalt core–shell nanoparticles on a h-terminated si (100) and by nanostructured cobalt-coated si nanowire electrodes. *ACS Appl. Mater. Interfaces* **5**(7), 2410–2416 (2013). 23488767 10.1021/am3021763

[64] Chen, X., Wang, X. & Fang, D. A review on C1s XPS-spectra for some kinds of carbon materials. *Fullerenes Nanotubes Carbon Nanostruct.* **28**(12), 1048–1058 (2020).

[65] Eltaweil, A. S. et al. Enhanced fenton degradation of tetracycline over cerium-doped MIL88-A/g-C3N4: Catalytic performance and mechanism. *Nanomaterials* **14**(15), 1282 (2024). 39120389 10.3390/nano14151282 PMC11313986

[66] Bulin, C. & Guo, T. Reaction thermodynamics of zerovalent iron and hexavalent chromium at the solid–liquid interface and in solution. *Langmuir* **40**(12), 6342–6352 (2024). 38483101 10.1021/acs.langmuir.3c03907

[67] Marjanović, V. et al. Adsorption of chromium (VI) from aqueous solutions onto amine-functionalized natural and acid-activated sepiolites. *Appl. Clay Sci.* **80**, 202–210 (2013).

[68] Abd El-Monaem, E. M., Omer, A. M. & Eltaweil, A. S. Durable and low-cost chitosan decorated Fe/MOF-5 bimetallic MOF composite film for high performance of the Congo red adsorption. *J. Polym. Environ.* **32**(5), 2075–2090 (2024).

[69] Ayoup, M. S. et al. Zwitterionic MOF-embedded alginate beads with polydopamine surface functionalization for efficient doxycycline removal: optimization and mechanistic study. *Int. J. Biol. Macromol.* **281**, 136288 (2024). 39368583 10.1016/j.ijbiomac.2024.136288

[70] Eltaweil, A. S. et al. Efficient removal of toxic methylene blue (MB) dye from aqueous solution using a metal-organic framework (MOF) MIL-101 (fe): Isotherms, kinetics, and thermodynamic studies. *Desalin. Water Treat.* **189**, 395–407 (2020).

[71] Eltaweil, A. S. et al. Fabrication of attapulgite/magnetic aminated chitosan composite as efficient and reusable adsorbent for cr (VI) ions. *Sci. Rep.* **11**(1), 16598 (2021). 34400760 10.1038/s41598-021-96145-6 PMC8368087

[72] Omer, A. M., Abd El-Monaem, E. M. & Eltaweil, A. S. Novel reusable amine-functionalized cellulose acetate beads impregnated aminated graphene oxide for adsorptive removal of hexavalent chromium ions. *Int. J. Biol. Macromol.* **208**, 925–934 (2022). 35364200 10.1016/j.ijbiomac.2022.03.187

[73] El-Nemr, M. A. et al. Adsorption of Cr6 + ion using activated Pisum sativum peels-triethylenetetramine. *Environ. Sci. Pollut. Res.* **29**(60), 91036–91060 (2022). 10.1007/s11356-022-21957-6 PMC9722890 35881295

[74] Eltaweil, A. S. et al. Engineering a sustainable cadmium sulfide/polyethyleneimine-functionalized biochar/chitosan composite for effective chromium adsorption: optimization, co-interfering anions, and mechanisms. *RSC Adv.* **14**(31), 22266–22279 (2024). 39010926 10.1039/d4ra03479a PMC11247309

[75] Niu, C. et al. Preparation of a novel citric acid-crosslinked Zn-MOF/chitosan composite and application in adsorption of chromium (VI) and methyl orange from aqueous solution. *Carbohydr. Polym.* **258**, 117644 (2021). 33593538 10.1016/j.carbpol.2021.117644

[76] Çelebi, H. Recovery of detox tea wastes: usage as a lignocellulosic adsorbent in Cr6 + adsorption. *J. Environ. Chem. Eng.* **8**(5), 104310 (2020).

[77] Dong, F. X. et al. Simultaneous adsorption of cr (VI) and phenol by biochar-based iron oxide composites in water: performance, kinetics and mechanism. *J. Hazard. Mater.* **416**, 125930 (2021). 34492860 10.1016/j.jhazmat.2021.125930

[78] Abd El-Monaem, E. M. et al. Construction of attapulgite decorated cetylpyridinium bromide/cellulose acetate composite beads for removal of cr (VI) ions with emphasis on mechanistic insights. *Sci. Rep.* **14**(1), 12164 (2024). 38806605 10.1038/s41598-024-62378-4 PMC11133475

[79] El-Nemr, M. A. et al. Fabrication of pea pods biochar-NH2 (PBN) for the adsorption of toxic Cr6 + ion from aqueous solution. *Appl. Water Sci.* **13**(10), 194 (2023).

[80] Feng, Y. et al. Fabrication of MXene/PEI functionalized sodium alginate aerogel and its excellent adsorption behavior for cr (VI) and Congo Red from aqueous solution. *J. Hazard. Mater.* **416**, 125777 (2021). 33839501 10.1016/j.jhazmat.2021.125777

[81] Zhang, H. et al. Chitosan-stabilized FeS magnetic composites for chromium removal: characterization, performance, mechanism, and stability. *Carbohydr. Polym.* **214**, 276–285 (2019). 30925998 10.1016/j.carbpol.2019.03.056

