# Supplementary material for: Innovative zero-valent cobalt decoration on MIL-88 A(Fe)@β-CD for high-efficiency and reusable cr(VI) removal
Source: Sci Rep. 2025 Feb 5;15:4316. doi: 10.1038/s41598-025-88259-y (PMC11799185; doi:10.1038/s41598-025-88259-y)

**Supplementary Information**

**Innovative Zero-Valent Cobalt Decoration on MIL-88A(Fe)@β-CD for High-Efficiency and Reusable Cr(VI) Removal**

**Eman M. Abd El-Monaem ^1^, Nouf Al Harby ^2,^*, Mervette El Batouti ^3^ and Abdelazeem S. Eltaweil ^3,4,*^**

^1^ Advanced Technology Innovation, Borg El-Arab, Alexandria, Egypt (E.M.A) ([emanabdelmonaem2014@gmail.com](mailto:emanabdelmonaem2014@gmail.com)).

^2^ Department of Chemistry, College of Science, Qassim University, Buraidah 51452, Saudi Arabia (N.F.A.) ([hrbien@qu.edu.sa](mailto:hrbien@qu.edu.sa)).

^3^ Department of Chemistry, Faculty of Science, Alexandria University 21934, Alexandria, Egypt, (M.E.) (Mervette.elbatouti@ alexu.edu.eg).

^4^ Department of engineering, College of Engineering and Technology, University of Technology and Applied Sciences, Sultanate of Oman, (A.S.E) ([abdelazeemeltaweil@alexu.edu.eg](mailto:abdelazeemeltaweil@alexu.edu.eg))

***** Correspondence: N.F.A. ([hrbien@qu.edu.sa](mailto:hrbien@qu.edu.sa)), A.S.E ([abdelazeemeltaweil@alexu.edu.eg](mailto:abdelazeemeltaweil@alexu.edu.eg))

**Text S1.** The used chemicals in the preparation of ZVCo-MIL-88A(Fe)@β-CD composite.

Ferric chloride hexahydrate (FeCl_3_.6H_2_O, > 97 %), sodium borohydride (NaBH_4_, 99 %) and sodium hydroxide (NaOH, 97 %) were purchased from Sinopharm Chemical Reagent. Fumaric acid (F. Acid, 99 %), beta-cyclodextrin (β-CD, 99 %) and cobalt chloride hexahydrate (CoCl_2_.6H_2_O, 99 %) were brought from Guangdong Guanghua Science and Technology. Methanol (> 99 %) and ethanol (Et-OH, > 99 %) were obtained from Alpha Chemika.

**Text S2.** Characterization tools to analyze the properties of ZVCo-MIL-88A(Fe)@β-CD.

The morphology, chemical composition, magnetism and surface charge of ZVCo, MIL-88A(Fe), β-CD, and ZVCo-MIL-88A(Fe)@β-CD were investigated using Fourier Transform Infrared (Frontier, PerkinElmer, FTIR), Scanning Electron Microscope (JEOL 7500F, SEM), X-Ray Photoelectron Spectroscopy (ESCALAB 250XI, XPS), Zeta Potential (Malvern, ZP), and X-Ray Diffraction (PANalytical, XRD).

**Table S1.** Equations of the applied adsorption kinetic models

| **Kinetic Model** | **Equation** |
| --- | --- |
| **Pseudo first order** | $lnln (Q_{e} -Q_{t})=lnln Q_{e}-(\frac{k_{1}}{2.303})t (1)$ |
| **Pseudo second order** | $\frac{t}{Q_{t}}=\frac{1}{k_{2}Q_{e}^{2}+ \frac{1}{Q_{e}}\left( t \right) (2)}$ |
| **Elovich model** | $Q_{t}=\frac{1}{\beta} ln \left( \alpha\beta\right)+ \frac{1}{\beta}$ ln (t) (3) |

Where, Q_t_ and Q_e_ are amounts of Cr^6+^ uptakes at time t and equilibrium, respectively. k_1_ and k_2_ are the rate constants of Pseudo first order and Pseudo second order, respectively. h is the initial adsorption rate constant. α and β are Elovich coefficients that represent the initial adsorption rate and the desorption coefficient, respectively, also related to the extent of surface coverage and activation energy for chemisorption.

**Table S2.** Equations of the applied adsorption isotherm models

| **Model** | **Equation** |
| --- | --- |
| **Langmuir** | $\frac{C_{e}}{Q_{e}}=\frac{1}{K_{L} q_{max}}+\frac{C_{e}}{q_{max}}$ (4) |
| **Freundlich** | $lnln Q_{e} =lnln K_{f} +\frac{1}{n}lnln C_{e}$(5) |
| **Temkin** | $Q_{e}=B lnK_{T}+Blnln C_{e}$ (5) |
| **D-R** | $Ln Q_{e}=Ln Q_{s}-K_{DR}\varepsilon^{2}$,$\varepsilon=RT Ln \left( 1+\frac{1}{C_{e}} \right)$ $(7$, 8) |

Where, Q_e_ and C_e_ are the adsorption capacity and the concentration of the un-adsorbed Cr^6+^ at equilibrium, respectively. Q_max_ and K_L_ are the monolayer adsorption capacity and Langmuir constant, respectively. n and K_f_ are Freundlich constants. K_T_ is the equilibrium binding constant and $B=\frac{RT}{b}$ , b is Temkin constant related to heat of adsorption. R is the gas constant (8.314 J/mol.k) and T is the absolute temperature. Q_s_ is the saturation capacity, ε is the Polanyi potential, K_DR_ is a constant related to the mean free energy of adsorption per mole of the adsorbate and E is the mean free energy of adsorption.

**Figure S1.** The linear Van't Hoff's curve of the Cr(VI) adsorption reaction onto ZVCo-MIL-88A(Fe)@β-CD.


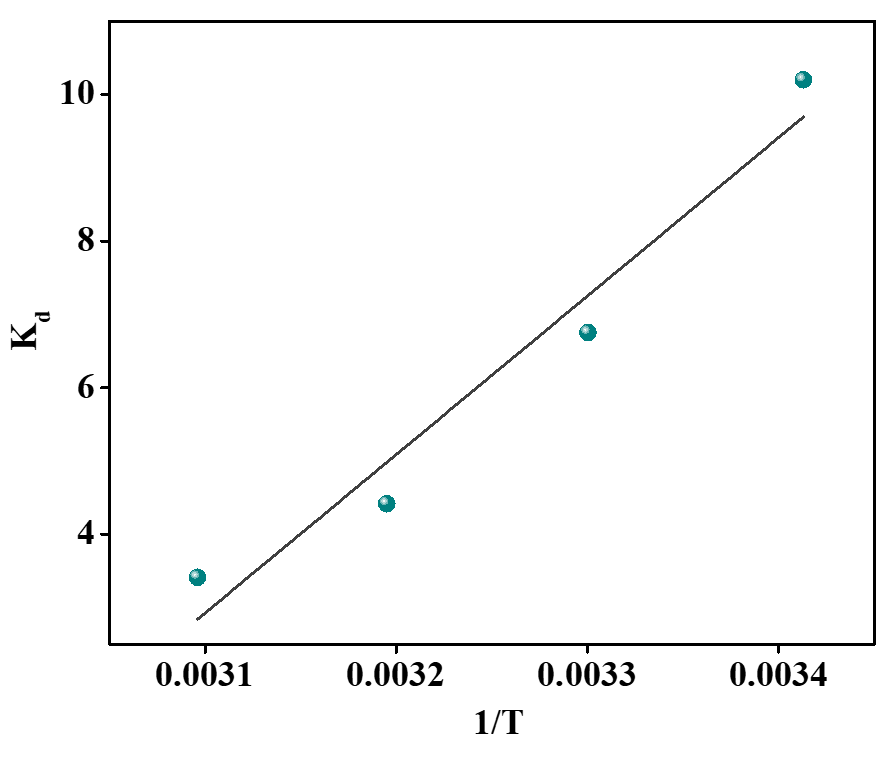

Supplement: Supplementary file 1 — Supplementary Material 1 [file 41598_2025_88259_MOESM1_ESM.docx]
